# NR2F6 promotes the malignant progression of neuroblastoma as an indicator of poor prognosis

**DOI:** 10.1371/journal.pone.0324334

**Published:** 2025-05-27

**Authors:** Yimeng Liu, Zhaoxia Zhang, Tao Mi, Liming Jin, Zhaoying Wang, Mujie Li, Jinkui Wang, Xin Wu, Junyi Luo, Jiayan Liu, Chunnian Ren, Dawei He

**Affiliations:** 1 Department of Urology, Children’s Hospital of Chongqing Medical University, National Clinical Research Center for Child Health and Disorders, Ministry of Education Key Laboratory of Child Development and Disorders, Chongqing Key Laboratory of Pediatrics, International Science and Technology Cooperation Base of Child Development and Critical Disorders, Chongqing, P.R China; 2 Chongqing Key Laboratory of Children Urogenital Development and Tissue Engineering, Chongqing, P.R China; 3 Department of pediatrics, The Second Clinical College of Chongqing Medical University: The Second Affiliated Hospital of Chongqing Medical University, Chongqing, P.R China; Zhejiang Cancer Hospital, CHINA

## Abstract

**Background:**

NR2F6 is an orphan nuclear receptor with dual tumorigenic activity in the immune system and tumor cells, playing an essential role in tumor differentiation and immunity. This study aimed to investigate the expression level of NR2F6 in various tumors and its effect on neuroblastoma (NB).

**Methods:**

We evaluated the role of NR2F6 in the genesis and development of 34 different tumors through multiple databases. In addition, we investigated the effects of NR2F6 expression levels on NB risk factors and prognosis using pathology sections and clinical data from primary retroperitoneal NB in children. The effects on cell proliferation, invasion, and migration were explored by knocking down NR2F6 expression in SK-N-BE(2) and SK-N-SH cells.

**Results:**

The findings showed that NR2F6 was significantly correlated with the prognosis of NB and was an important indicator suggesting disease regression. In addition, NR2F6 knockdown slowed down NB cells’ proliferation, invasion, and migration ability in vitro.

**Conclusion:**

Our results suggest that NR2F6 plays a crucial role in tumor-promoting effects and can be used as a potential prognostic marker for NB.

## Introduction

Cancer is currently the most significant life-threatening public health problem encountered by humankind. According to a report by the World Health Organization, about 9.6 million people died of cancer worldwide in 2018 (https://www.who.int/news-room/fact-sheets/detail/cancer). Cancer treatment has been plaguing the healthcare system. Due to individual differences, patients have varying sensitivities to treatments. Thus, finding high-coverage or targeted treatments has become our new goal, and immunotherapy is gradually becoming a new direction in cancer treatment as research continues to deepen [1].

Advancements in sequencing technology and bioinformatics have enabled us to identify tumor driver genes, molecular subtypes, molecular interactions, and other features using large-scale cancer genomics databases such as The Cancer Genome Atlas (TCGA) and the International Cancer Genome Consortium (ICGC). Since cancer results from aberrant cell proliferation, differentiation, and immune stress, understanding the characteristics, alteration pathways, and immune properties of cancer’s unique tissue environment is significant for identifying potential targets for tumorigenesis and development [2].

NR2F6 (nuclear receptor subfamily 2F group member 6, also known as Ear-2) is an orphan nuclear receptor with dual pro-tumorigenic activity in the immune system and tumor cells [3]. NR2F6 might be a critical immune checkpoint in the T-cell compartment, controlling tumor progression and growth. Another immune cell type in which NR2F6 has been shown to play an important role is the macrophage, which may suppress immune cell genes in reverse transcriptional activation in human macrophages to promote tumor development [4]. NR2F6 has been confirmed in a variety of tumors have promoted the development of tumors, such as in ovarian breast, cervical, colon, hepatocellular, and non-small-cell lung cancers [5–9]. NR2F6 is a prospective biomarker, for tumor cells and long-term hematopoietic stem cells (LT-HSC) proliferation, metastasis, survival ability plays an important role, and can inhibit the differentiation process of leukemia cells [10]. Given the dual pro-tumorigenic activity of NR2F6 in immune cells and tumor cells, inhibition of NR2F6 expression offers a unique therapeutic potential to improve current treatment outcomes. With current studies demonstrating increased expression of NR2F6 in multiple cancer types, targeting NR2F6 could serve as an independent and potentially synergistic option. Gene modulation therapy, once feasible, may help promote local effector T cell responses at the tumor site with fewer systemic immune-related adverse events. Any rational drug design targeting NR2F6-mediated gene regulation would have critical clinical applications by affecting both tumor-abnormal effector T cells and tumor cells while inducing tumor cell death. Thus, the relationship between the level of NR2F6 expression in pancancer and immune infiltration and tumor prognosis deserves further investigation.

In this study, we comprehensively explored the expression of NR2F6 in 34 human cancers based on TCGA, TARGET, and GTEx analysis databases. We also evaluated the prognostic value of NR2F6 in pancancer and analyzed the relationship between the expression level of NR2F6 and immune cell infiltration, immune checkpoints, and immune regulatory genes, as well as the relationship between the expression level of NR2F6 and mutations (including TMB and MSI). Neuroblastoma (NB), originated from neural crest cells in the sympathetic nervous system, is the most common extracranial malignant tumor in children, accounting for 6% -10% of the incidence of pediatric tumors, with an incidence of about 1/100,000 [11]. Despite significant advances in therapy, the 5-year survival rate of high risk NB remains below 40%, with an extremely poor prognosis [12]. Immune-related therapies have been great success in a range of adult cancers, such as immune-checkpoint consistent (ICI) and adoptive T cell therapy. However, NB has low mutation load and low immunogenicity, and the lack of tumor infiltration of lymphocytes leads to suboptimal response to immunotherapy [13,14]. Given the dual tumorigenic activity of NR2F6 in immune cells and tumor cells, inhibition of NR2F6 expression offers a unique therapeutic potential to improve current therapeutic outcomes. Therefore, we paid special attention to the differential expression of NR2F6 in NB and its relationship with prognosis. We combined the gene expression of NB with clinical data, and initially explored the role of NR2F6 on NB at the cellular level and in clinical samples, so as to provide new potential targets for the diagnosis and treatment of NB.

## Materials and methods

### Data extraction

We extracted expression data from the UCSC (https://xenabrowser.net/) database (accessed on May 24, 2023), including TCGA, TARGET, and GTEx (PANCAN, N = 19131, G = 60499). We further extracted the expression data of the ENSG00000160113 (NR2F6) gene in each sample. We obtained expression data and clinical information for 34 cancer types, and we performed a log2(x + 0.001) transformation for all expression value. We obtained a high-quality prognostic dataset for TCGA from a previous TCGA prognostic study published in Cell and a high-quality prognostic dataset for TCGA from the Cancer Browser at UCSC (https://xenabrowser.net/datapages/), supplemented with TARGET follow-up data and excluded samples with a follow-up time shorter than 30 days [15]; furthermore, a log2(x + 0.001) transformation was performed for each expression value, and finally, we also excluded cancers with less than 10 samples in single type of cancer to obtain the prognostic data.

### Pancancer analysis of NR2F6 expression

We calculated the expression differences between normal and tumor samples in each type of tumor using R software (version 3.6.4), and the significance of differences was analyzed using unpaired Wilcoxon Rank Sum and Signed Rank Tests. To investigate the relationship between NR2F6 expression and clinicopathological features in multiple cancers, we evaluated NR2F6 expression in patients with stage I, II, III, and IV in the TCGA database by the R software (version 3.6.4). We used unpaired Student’s t-test for a two-by-two analysis of the significance of differences and ANOVA to test the differences of samples in multiple groups.

### Prognostic analysis

We used the Coxph function of the R package survival (version 3.2–7) to build a Cox proportional hazards regression model to analyze the significance of the NR2F6 gene in predicting overall surviva l(OS), disease-specific survival (DSS), disease-free interval (DFI) and progression-free interval (PFI) in pancancer and statistical tests were performed using Logrank test to obtain the prognostic significance and to draw forest map [16]. Furthermore, we examined the relationship between NR2F6 expression and OS in multiple cancer patients using Furthermore, we examined the relationship between NR2F6 expression and OS in multiple cancer patients using Kaplan-Meier curves.

### Analysis of the correlation between NR2F6 expression and tumor immunity

We extracted the expression data of the NR2F6 gene and 60 genes of the 2 types of immune checkpoint pathways Inhibitory (24), Stimulatory (36), and marker genes derived from the literature in individual samples [17]. Next, we calculated the Pearson correlation between NR2F6 and the marker genes of the five classes of immune pathways.We extracted the expression data of the NR2F6 gene and 150 marker genes of five types of immune pathways (chemokine (41), receptor (18), MHC (21), Immunoinhibitor (24), Immunostimulator (46) in individual samples, and calculated the Pearson correlation between NR2F6 and the marker genes of the five immune pathways.

We extracted gene expression profiles from them for each tumor separately and mapped the expression profiles to the GeneSymbol, and using the Timer method of the R package IOBR (version 0.99.9, https://www.ncbi.nlm.nih.gov/) [18], and the DECONVO_CIBERSOR method to perform a reliable immune score assessmens [19] (*p < 0.05,**p < 0.01, ***p < 0.001,****p < 0.0001).

### NR2F6 expression and gene mutation correlation analysis

We also downloaded the Simple Nucleotide Variation dataset at level 4 of all TCGA samples processed by MuTect2 software from GDC (https://portal.gdc.cancer.gov/) [20]. We integrated the mutation data of the samples and obtained the structural domain information of the proteins from the R software package map tools (version 2.2.10). We evaluated the mutations using the chi-square test. We used chi-square tests to assess differences in the frequency of mutations in each group of samples.

### Multifactor Cox regression analysis and construction of nomogram

In this study, we evaluated the independent risk factors for NR2F6 in 151 samples from the Target-NB queue, using the R package survival, integrating data on survival time, survival status, eight characteristics, and gene expression of NR2F6 by the Cox regression method. Based on the multifactorial Cox regression analyses, we performed ROC analyses to obtain the AUC using the R package pROC (version 1.17.0.1). Using the R software package rms, data on survival time, survival status, and three characteristics were integrated, and a nomogram was built using the Cox method to assess the prognostic significance of these characteristics in 151 samples.

### Clinical data statistics

We retrospectively included children with primary retroperitoneal NB who underwent surgical management at the Children’s Hospital of Chongqing Medical University from January 2017 to May 2023. All studies involving human tissues were conducted in strict accordance with the Declaration of Helsinki and were approved by the Ethics Committee of Chongqing Medical 0 Nianlun Audit (Research) No. (170). Ethical approval has been uploaded as required. This study involved cases of children under the age of 14, all of whom had obtained the informed consent of their legal guardians. All participants were informed and consented to sample collection, intended research, and publication usage. Written consent was collected according to the ethical regulations of the Children’s Hospital of Chongqing Medical University.

Exclusion criteria were no surgical treatment, without patient consent, and lack of adequate tumor tissue. 61 patients largely met the inclusion criteria. We reviewed the medical history, surgical details, histology, tumor staging, and postoperative data. Statistical analysis was performed using IBM SPSS 25.0 window statistics (SPSS Statistics, Statistics 25, IBM Corporation, Armonk, NY, USA). We used chi-square for univariate and NR2F6 simple correlation analysis. Survival data multivariate analyses were performed using multifactorial logistic regression and multifactorial Cox regression. P < 0.05 was considered to be different.

### Immunohistochemical staining

Tissue sections of NB were provided by the Department of Pathology, Affiliated Children’s Hospital of Chongqing Medical University. Paraffin sections were examined for NR2F6 expression levels in NB tissues using immunohistochemical square staining, detailed as described in previous work [21]. The stained sections were then scanned using a TEKSQRAY slide scanning imaging system (Biostrong Technology, China), after which the slides were evaluated for NR2F6 expression by an experienced pathologist. Five high-magnification fields of view (×400) were randomly observed on each slide. They averaged, and the scoring criteria were based on the German immunohistochemical score (GIS) [22]: (1) Staining intensity: no staining of the cells, 0 points; light yellow, 1 point; medium yellow or brown, 2 points; dark brown or tan, 3 points; (2) Proportion of positive cells: no positive cells, 0 points; < 25%, 1 point; less than 50%, 2 points; < 75%, 3 points; > 75%, 4 points. Finally, the two scores were added together: 0–3: low expression; 4–7: high expression.

### Cell lines and cell culture

NB cell line SK-N-SH (MYCN non-amplification), SK-N-BE (2) cell (MYCN amplification), and HEK-293 (human embryonic kidney cells) were purchased from the cell bank of the Chinese Academy of Sciences (Procell Life Science&Technology Co., Ltd., China). SK-N-DZ(MYCN amplification) was purchased from the American Type Culture Collection (ATCC, Manassas, VA, USA). All cells were cultured using DMEM medium (MeilunBio, MA0212, Dalian, China) adding 10% serum (GIBCO fetal bovine serum, New Zealand) and 1% antibiotics (100 mg/mL streptomycin and 100 U/mL penicillin). The cell incubator was set up at 37°C with 5% CO_2_.

### Cell transfection and RNA sequencing

Negative control (NC) siRNA and siRNA targeting NR2F6 were purchased from Qingdao MDBio, Inc.(China).6-well cell culture plates were inoculated with 3 × 10^5^ SK-N-BE (2) and SK-N-SH cells per well. Based on the transfection of this oligomer into SK-N-BE (2) and SK-N-SH cells according to the manufacturer’s protocol, using Lipofectamine 2000 reagent (Invitrogen, CA, USA) [5]. 5μM of Si-NR2F6 3–1, Si-NR2F6 3–2, Si-NR2F6 3–3, and the corresponding controls (Genepharma, Shanghai, China) were transfected into cells. After 6 hours of transfection, the medium was changed to a complete medium, and the cells were cultured until 48 hours. Cells were collected, and the knockdown efficiency of NR2F6 was detected by western blot and qPCR, respectively. The RNA sequences were as follows in S1 Table. In addition, to screen for downstream pathways regulated by the NR2F6 gene, six samples of NR2F6 downregulated SK-N-BE (2) cells and control SK-N-BE (2) (three for each) were subjected to to high throughput sequencing of mRNA(Shanghai Majorbio Technology Co., LTD). Differentially expressed genes were screened by RNA sequencing data and differential genes were displayed using volcano plots and heatmaps. KEGG functional enrichment analysis was also performed for differential genes, and Single-gene GSEA enrichment analysis for NB was performed using the reactome dataset. The details is described in previous work [23].

### qRT-PCR

RNA from HEK293, SK-N-SH, SK-N-DZ and SK-N-BE (2) cells was extracted for reverse transcription and qRT-PCR to detect mRNA expression levels of NR2F6 gene as described in previous work [20]. We used the expression level of GAPDH mRNA as a reference [18]. qRT-PCR analysis primers were synthesized by Sango Biotech (Shanghai, China) as follows in S2 Table.

### Western blot

The levels of NR2F6 protein expression as well as key proteins of the MAPK signaling pathway were measured by Western blot, with specific steps described in previous work [24]. Antibody information including NR2F6, p-JNK, JNK, p-ERK, ERK, respectively, p-p38, p38, and GAPDH are provided in S3 Table.

### Cell proliferation, cell cycle, as well as invasive migration ability detection

The proliferation, migration and invasion abilities of NB cells were examined using CCK-8 assay, cell scratch and Transwell methods, respectively, with reference to previous work [20].

### Statistical analysis

Data were analyzed and statistical charts were created using SPSS 25.0 and the graphics board Prism 8. All measurements were expressed as mean ± standard deviation (mean ± SD) and comparisons between two groups were performed using the t-test. Comparisons between multiple groups were performed using ANOVA. The rank-sum test was used for the ranked data. P < 0.05 was considered to be statistically significant.

## Results

### Analysis of the presence of differential expression of NR2F6 in pancancer tissues

**Fig 1:**
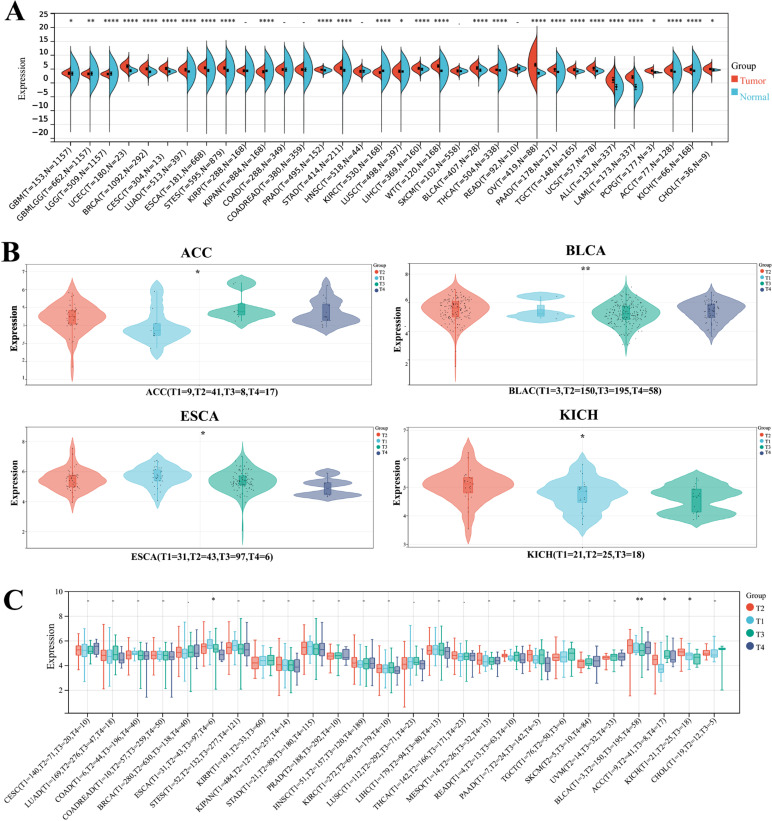
Differential expression of NR2F6 in pancancer and differential expression in clinical stages. A:Differential expression of NR2F6 in tumor tissues and adjacent normal tissues of pancancer (*P < 0.05; **P < 0.01; ***P < 0.001; ****P < 0.0001). B:Differential expression of NR2F6 in different clinical stages of pancancer(*P < 0.05; **P < 0.01).

We obtained expression data for 34 cancer types(The full English name of cancer abbreviation is given in S4 Table. Significant upregulation of NR2F6 mRNA levels was observed in 25 tumors: GBM, GBMLGG, UCEC, BRCA, CESC, LUAD, ESCA, STES, PRAD, STAD, LUSC, LIHC, WT, BLCA, THCA, OV, PAAD, TGCT, UCS, ALL, LAML, PCPG, ACC, KICH, CHOL: we observed significant downregulation in 3 tumors such as LGG, KIPAN, KIRC (Fig 1A). Our results showed that NR2F6 expression was significantly upregulated in human cancers relative to adjacent normal tissues.

### Expression levels of NR2F6 at different clinical stages in pancancer

By analyzing the expression of 26 cancer types, we observed significant differences in NR2F6 expression levels in four tumors (ESCA, BLCA, ACC, KICH) in different clinical stages (Fig 1B).

### Correlation analysis between NR2F6 expression level and prognosis of pancancer

**Fig 2:**
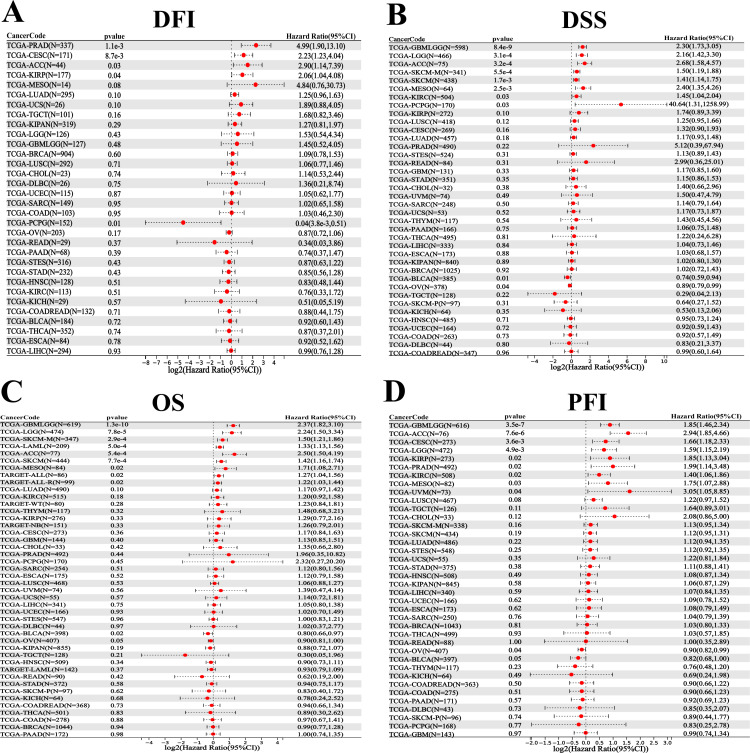
Forest plot showing the relationship between NR2F6 expression and pancancer prognosis. A:Association between NR2F6 expression and DFI in pancancer. B:Association between NR2F6 expression and DSS in pancancer. C: Association between NR2F6 expression and OS in pancancer. D: Association between NR2F6 expression and PFI in pancancer. The red color represents significant results.

**Fig 3:**
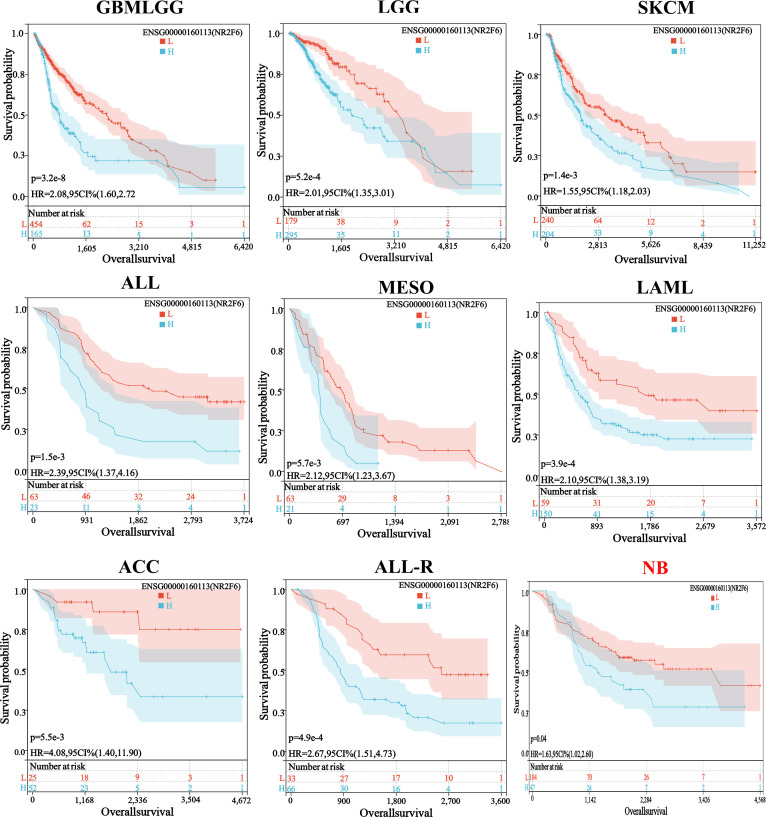
Kaplan-Meier curves of OS in high NR2F6 expression and low NR2F6 expression groups in glioma (GBMLGG), low-grade glioma (LGG), small-cell lung cancer (SKCM), acute lymphoblastic leukemia (ALL), malignant mesothelioma (MESO), acute myeloid leukemia (LAML), and acute lymphoblastic leukemia-type R (ALL-R), neuroblastoma (NB).

We obtained expression data for 32 cancer types and DFI data for the corresponding samples and observed that higher expression of NR2F6 in CESC, KIRP, PRAD and ACC was associated with worse DFI, and higher expression of NR2F6 in the PCPG was associated with better DFI (Fig 2A). Expression data for 38 cancer types were obtained as well as DSS data from corresponding samples, and higher expression of NR2F6 in GBMLGG, LGG, KIRC, SKCM, SKCM, CM-M, MESO, PCPG, and ACC was associated with worse DSS, and higher expression in BLCA and OV was associated with better DSS (Fig 2B). The expression data of 44 cancer types and OS data of corresponding samples were obtained, and higher NR2F6 expression was associated with worse OS in GBMLGG, LGG, SKCM, ALL, MISO, LAML, ACC, ALL-R, and NB, and higher expression of NR2F66 in BLCA was associated with better OS (Fig 2C). The Kaplan-Meier survival curve showed that the high NR2F6 expression group showed poor OS in GBMLGG, LGG, SKCM, ALL, MESO, LAML, ACC, ALL-R, and NB tumors (Fig 3). In expression data from 38 cancer types and PFI data from corresponding samples, we observed higher expression of NR2F6 in GBMLGG, LGG, CESC, KIRP, CESC, KIRP, KIRP, KIRC, MESO, UVM, ACC, and higher expression of NR2F6 in BLCA and OV with better PFI (Fig 2D).

### Expression and immune correlation analysis of NR2F6

**Fig 4:**
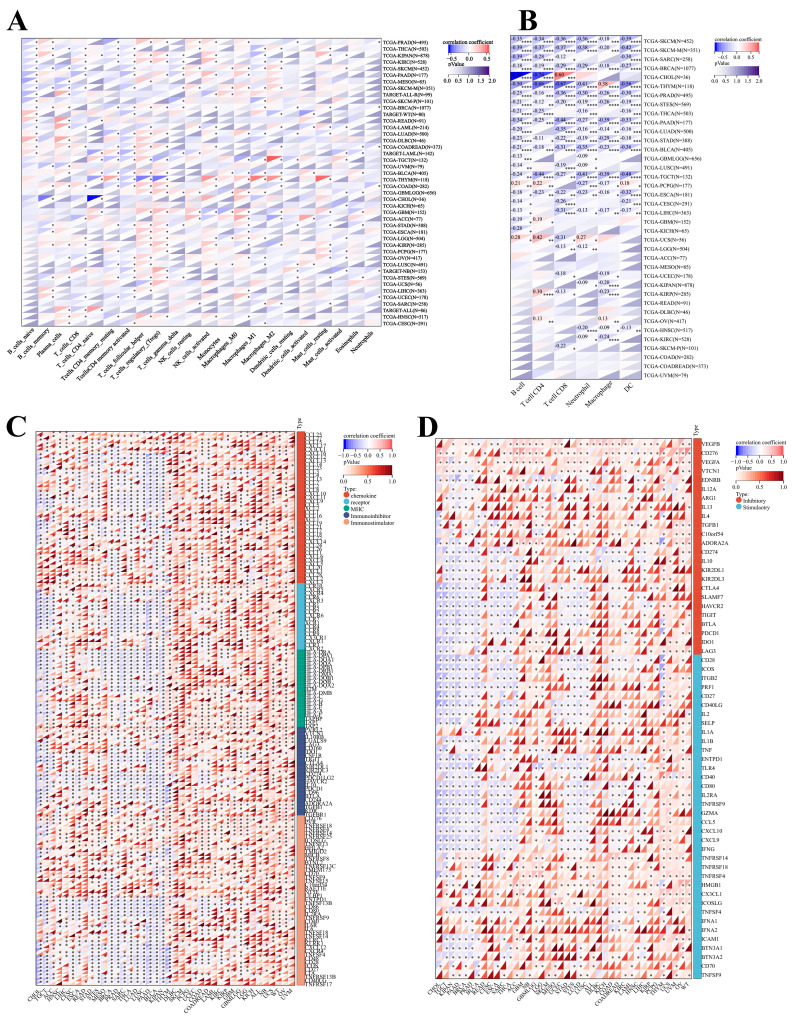
Correlation analysis of NR2F6 expression levels with immune cell infiltration, immune checkpoints, and immunomodulatory genes in pancancer. A: The expression level of NR2F6 was significantly correlated with the infiltration level of various immune cells based on the CIBERSORT algorithm in pancancer. B: NR2F6 expression was significantly correlated with the infiltration levels of various immune cells based on the timer algorithm in pancancer.* p < 0.05, * * p < 0.01, * * * p < 0.001, and * * * * p < 0.0001. C: Correlation between the NR2F6 expression level and the expression level of the immune checkpoint genes in pancancer. D: Correlation between NR2F6 expression level and expression levels of immune regulatory genes in pancancer. *P < 0.05.

Based on the CIBERSORT method, we observed that NR2F6 expression was significantly associated with 22 classes of immune cell infiltration in 44 cancer types. We focused on the expression of NR2F6 and the immune cell infiltration in NB. In NB, NR2F6 expression was significantly positively correlated with CD8 + T cells, T_cells_regulatory_(Tregs) and Dendritic_cells_resting cell infiltration, but it was significantly negatively associated with T _ cells _ CD _ 4 _ memory _ activated, master cell activation, and immune cell infiltration such as neutrophils (Fig 4A). Based on the TIMER method, we observed that NR2F6 expression was significantly associated with immune infiltration in 31 cancer types: B cells in 23 types of cancer, CD4 + T cells in 19 types of cancer, CD8 + T cells in 22 types of cancer, neutrophils in 22 types of cancer, macrophages in 20 types of cancer, and dendritic cells in 18 types of cancer. (Fig 4B).

We analyzed the relationship between NR2F6 expression and immune regulatory genes in different cancer types. The Pearson correlation analysis of NR2F6 and immunoregulatory genes showed that NR2F6 expression significantly negatively correlated with immunoregulatory genes in several cancers including KIPAN, TGCT and BRAC, and NR2F6 expression significantly positively correlated with expression of most immunoregulatory genes in most cancers. Furthermore, we observed that in NB NR2F6 expression was significantly positively correlated with the expression of almost all immunoregulatory genes (Fig 4C). In addition, we also analyzed the relationship between the expression level of NR2F6 and the immune checkpoint (ICP) genes. We calculated the Pearson correlation coefficient between NR2F6 expression and 60 immune checkpoint genes, including 24 suppressor genes and 36 stimulated genes. The results observed that in most tumors, immune checkpoint genes were significantly positively correlated with NR2F6 expression, while in some cancers, such as CHOL, KIPAN, BRCA, TGCT, PAAD, most immune checkpoint genes were significantly negatively correlated with NR2F6 expression, especially genes such as CTLA4, SLAMF7, HAVCR2 and TIGIT. However, in NB, NR2F6 expression was significantly and positively correlated with the expression of immune checkpoint genes such as TNFSF9, HMGB 1, TNFRSF4, TNF, PRF 1, LAG 3, IDO 1, VE GF B, CD276, VE GF A, and AL12A (Fig 4D).

### Correlation analysis of NR2F6 expression levels and mutational landscape in pancancer

**Fig 5:**
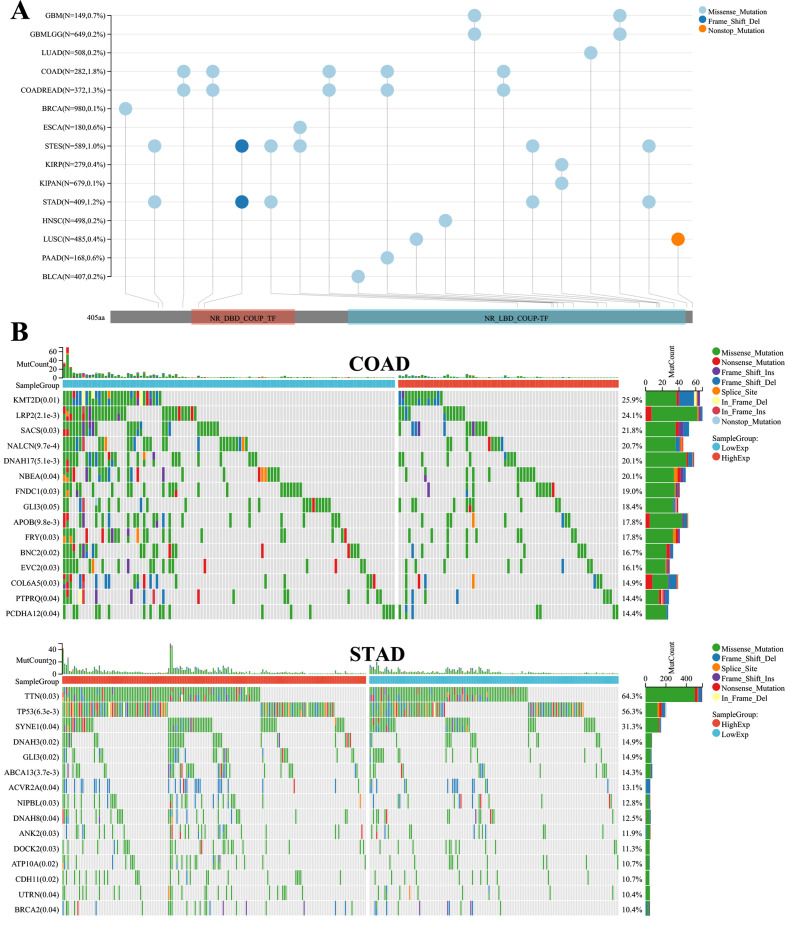
Landscape mutations of NR2F6 in different pancancers. A:Landscape of NR2F6 mutations in 15 cancer types. B: Landscape of NR2F6 mutations in COAD and STAD.

The mutational landscape suggested that NR2F6 could be observed as a distinct gene mutation in cancers such as colon cancer (COAD) and gastric adenocarcinoma (STAD) (Fig 5A). Therefore, we focused on analyzing the relationship between NR2F6 expression and specific genomic features, such as somatic mutations and copy number variations (copy number variants) in the COAD and STAD data. In COAD, 288 samples were tested for mutations, of which the mapping samples contained 173 (60.1%), and the top 10 genes in terms of mutation rate included KMT2D, LRP2, and SACS. There were significant differences in the mutation rate between the high and low NR2F6 expression groups. In STAD, 414 samples were detected with mutations, of which the mapping samples contained 335 (80.9%), and the top 10 genes with mutation rates included TTN, TP53, SYNE1, etc. There was a significant difference in mutation rates between NR2F6 high-expression and low-expression groups (Fig 5B).

### Construction of a nomogram affecting pediatric OS in the Target-NB cohort

**Fig 6:**
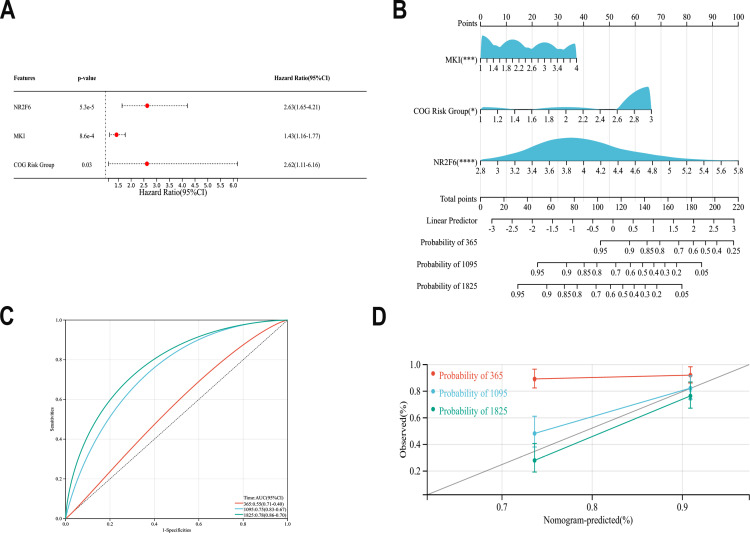
The Target-NB data was used to screen risk factors affecting NB prognosis and to construct nomograms. A:Multifactorial Cox analysis affecting OS in patients with NB. B:Nomogram for predicting OS in children with NB. C:ROC curve analysis assesses the accuracy of the nomogram. D:calibration curves for the nomogram used to predict 1-, 3-, and 5-year survival, The probability of survival predicted by the nomogram is plotted on the x-axis; the actual survival rate is plotted on the y-axis.

Using data from the Target-NB cohort, combining NR2F6 expression level and clinicopathological factors, using multivariate Cox regression analysis of risk factors affecting OS in NB children, the independent risk factors affecting OS in NB patients were MKI, COGRisks and NR2F6 expression (Fig 6A). A nomogram was constructed based on the above independent risk factors to predict the OS of NB patients (Fig 6B). The C index of the prediction model was 0.696(95% CI0.63–0.75), indicating that the prediction model of the nomogram has good discriminatory ability. The discriminatory ability of the column charts was tested by AUC, with an AUC of 90.0,81.0 and 76.0, respectively (Fig 6C). The calibration curve shows that the predicted value of the column chart is highly consistent with the actual observed value, indicating that the nomogram has good accuracy(Fig 6D).

### NR2F6 expression is associated with NB prognosis

**Fig 7:**
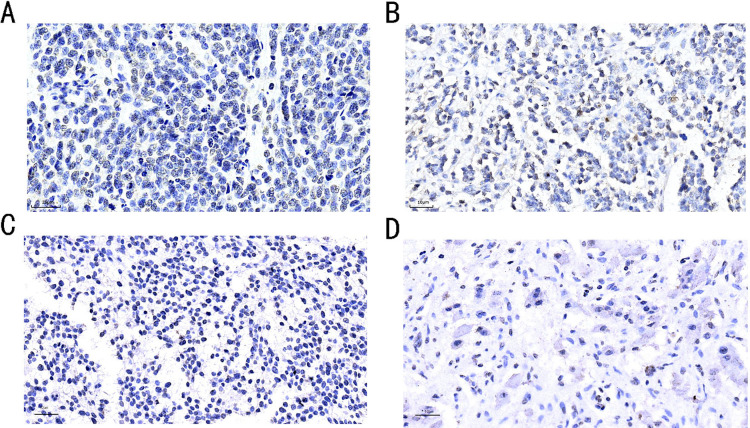
The expression level of NR2F6 in NB tissues was determined by IHC. A: INSS Stage 4, COG:High-risk, uFH, GIS:6, None-Differentiated Neuroblastoma,x400; B: INSS Stage 3, COG:Poor-risk, uFH, GIS:5, Differential Neuroblastoma,x400; C: INSS Stage 1, COG:Low-risk, FH, GIS:4, Poorly-Differentiated Neuroblastoma,x400; D: INSS Stage 1, COG:Low-risk, FH, GIS:2, Arthroblastoma, × 400).

We selected 61 children with retroperitoneal NB from Chongqing Children’s Hospital for immunohistochemical staining of pathological tissues. We found that the expression of NR2F6 in pathological sections of children with different levels of risk and pathologic staging grades varied and might be associated with prognosis. There is no verifiable threshold because there is no validated information on the evaluation or significance of NR2F6 staining in NB. We converted the mean value of staining intensity to a GIS score, with 0–3 being low expression and 4–7 being high expression. The distribution of NR2F6 staining intensity is shown in (Fig 7).

We analyzed the clinical data based on the GIS score of NR2F6, as shown in Table 1. The one-way chi-square test suggested no significant difference between the low and high NR2F6 GIS expression groups in terms of age, gender, and prognostic type (P > 0.05). INSS grading (c 2 = 19.981, P < 0.001, COG score (c 2 = 14.866, P = 0.001), MYCN gene expression (c 2 = 10.524, P = 0.001), degree of tissue differentiation (c 2 = 8.907, P = 0.031), number of tissue metastases (c 2 = 12.608, P = 0.027), bone marrow metastasis (c 2 = 10.370, P = 0.001), and survival status (c 2 = 31.878, P < 0.001) were statistically different between the low and high NR2F6 GIS expression groups.

**Table 1:** Univariate correlation analysis of NR2F6 gene and clinically relevant risk factors in children with NB.

Variable	N = 61	NR2F6 Low (N = 39)	NR2F6 High (N = 22)	χ^2^	*P*
Age:				7.007	0.328
<12m	18(29.5%)	14(77.8%)	4(22.2%)		
12-18m	7(11.5%)	4(57.1%)	3(42.9%)		
>18m	36(59.0%)	21(58.3%)	15(41.7%)		
Gender:				0.766	0.382
Male	35(57.4%)	24(68.6%)	11(31.4%)		
Female	26(42.6%)	15(57.7%)	11(42.3%)		
INSS:					
1	12(19.7%)	12(100%)	0	19.981	<0.001
2	3(4.9%)	2(66.7%)	1(33.3%)		
3	10(16.4%)	9(90%)	1(10%)		
4	36(59%)	16(44.4%)	20(55.6%)		
COG:				14.866	0.001
Low-risk	13(21.3%)	13(100%)	0		
Poor-risk	10(16.4%)	7(70%)	3(30%)		
High-risk	38(62.3%)	19(50%)	19(50%)		
MYCN:				10.524	0.001
Non-amplify	36(59.0%)	29(80.6%)	7(19.4%)		
Amplify	25(41.0%)	10(40%)	15(60%)		
Differentiated:				8.907	0.031
None	2(3.3%)	1(50%)	1(50%)		
Differential	40(65.6%)	25(62.5%)	15(37.5%)		
Poorly	11(18.0%)	5(45.5%)	6(54.5%)		
Mixed	8(13.1%)	8(100%)	0		
Shimada:				2.086	0.149
FH	21(34.4%)	16(76.2%)	5(23.8%)		
uFH	40(65.6%)	23(57.5%)	17(42.5%)		
Organ metastasls:					
0	34(55.7%)	28(82.4%)	6(17.6%)	12.608	0.027
1	15(24.6%)	6(40%)	9(60%)		
2	3(4.9%)	1(33.3%)	2(66.7%)		
3	3(4.9%)	1(33.3%)	2(66.7%)		
4	3(4.9%)	1(33.3%)	2(66.7%)		
5	3(4.9%)	2(66.7%)	1(33.3%)		
Marrow metastasls:				10.370	0.001
No	43(70%)	33(76.7%)	10(23.3%)		
Yes	18(30%)	6(33.3%)	12(66.7%)		
Living condition:				31.878	<0.001
Alive	37(60.7%)	34(91.9%)	3(8.1%)		
Death	24(39.3%)	5(20.8%)	19(79.2%)		

NR2F6 expression level was assessed by immunohistochemical score and German immunohistochemical score (GIS), 0–3: low expression; 4–7: high term. Organ metastasis is the cumulative number of organ metastasis. The final statistical results were considered to be credible with P < 0.05.

We used multivariate logistic regression and multivariate Cox regression to evaluate the effect of NR2F6 with other factors on patient OS, and the results are shown in Table 2. The results showed the significant effect of COG risk score, number of tissue metastasis and NR2F6 expression on OS in children with NB (OR = 32.46,95% CI 5.62–187.29, P = 0.006).

**Table 2:** Multivariate logistic regression analysis of NB disease outcome with NR2F6 gene levels and other risk factors.

Variable	b	S.E	OR	OR(95%CI)	*P*
Age:					
<18m					
≥18m	-1.072	0.471	0.342	0.294-1.359	0.240
Gender:					
Male					
Female	-0.594	0.625	0.552	0.162-1.880	0.324
INSS:					
1					
2/3/4	-0.625	0.696	0.535	0.137-2.095	0.369
COG:					
Low-risk					
Poor-risk					
High-risk	3.724	1.309	41.412	3.185-538.410	0.004
MYCN:					
Non-amplify					
Amplify	0.676	0.587	41.412	0.623-6.214	0.249
Differentiated:					
None					
Differential					
Poorly	0.681	0.435	1.975	0.842-4.631	0.117
Mixed					
Shimada:					
FH					
uFH	-0.982	0.846	0.375	0.71-1.965	0.246
Organ metastasls:					
0					
1/2/3/4/5	0.489	0.177	1.630	1.153-2.305	0.006
Marrow metastasls:					
No					
Yes	-0.291	0.551	0.748	0.254-2.202	0.598
NR2F6:					
Low					
High	3.480	0.894	32.462	5.624-187.291	<0.001

We included statistically significant univariate factors further in the multivariate regression analysis and critical information that might affect survival in multifactorial Cox regression to exclude confounding factors. We established a multifactorial Cox proportional risk model to predict the regression of the children’s disease, the results are presented in Table 3. Age, COG score, MYCN gene expression, tumor tissue differentiation, and NR2F6 expression were included to construct multifactorial linear regression equations. The results showed that the COG score (high versus low score) (P = 0.009) and the expression status of NR2F6 (high compared to low expression) had a statistically significant effect on OS in children with NB (P < 0.001).

**Table 3:** Multifactor Cox proportional risk model for survival analysis.

Variable	b	S.E	t	*P*
Age	-0.028	0.063	-0.448	0.656
COG	0.191	0.070	0.319	0.009
MYCN	0.039	0.105	0.369	0.714
Differentiated	0.049	0.067	0.724	0.472
NR2F6	0.605	0.101	5.964	<0.001

### In vitro, the knockdown of NR2F6 inhibits NB cell proliferation, invasion, and migration

**Fig 8:**
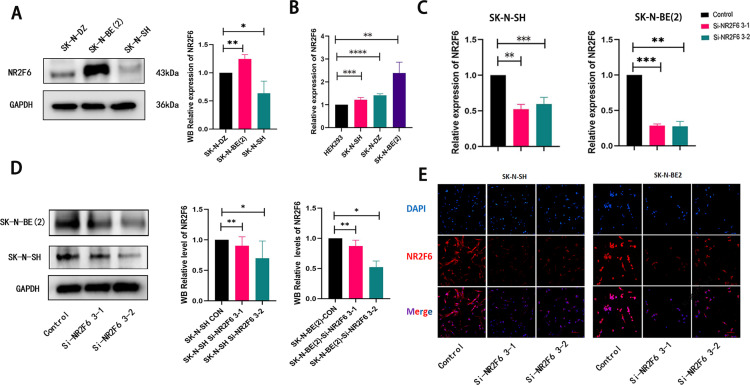
Validation of NR2F6 expression level and knockdown efficiency in NB cells. A: Protein expression levels of NR2F6 in several NB cell lines. B:mRNA expression levels of NR2F6 in several NB cell lines. C:The knockdown efficiency at the NR2F6 mRNA level in NB cells was verified by qPCR. D:The knockdown efficiency of NR2F6 protein levels in NB cells was verified by Western blot assay. E:The knockdown efficiency of NR2F6 in NB cells was verified by immunofluorescence. * P < 0.05, ** P < 0.01, *** P < 0.001, and **** P < 0.0001.

To investigate the biological function of NR2F6 in NB progression, we further performed related functional experiments at the cellular level. We first examined the expression level of NR2F6 in 3 different NB cell lines. The results showed that the protein expression levels and mRNA expression levels of NR2F6 were significantly higher than SK-N-SH and SK-N-DZ in SK-N-BE (2) cells (Fig 8A, 8B). SK-N-BE (2) (MYCN amplification NB cell) and SK-N-SH (MYCN non-amplification NB cell) were further selected for functional experiments. The results of RT-q-PCR, western blot and immunofluorescence suggested that si-NR2F6 3–1 and si-NR2F6 3–2 successfully downregulated the NR2F6 expression levels in both cells (Fig 8C, 8D, 8E).

**Fig 9:**
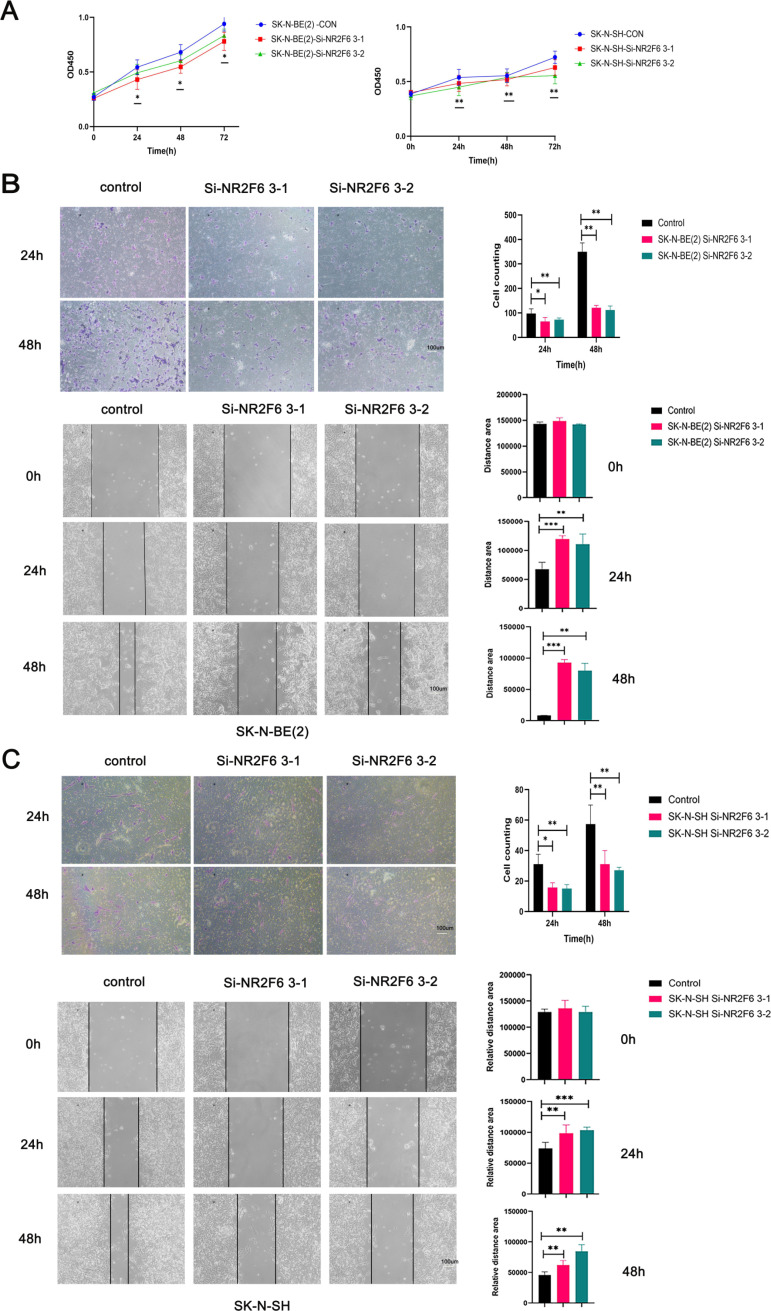
NR2F6 downregulation inhibits proliferation, migration and invasion of NB cells. (A) SK-N-BE(2) and SK-N-SH cell proliferation ability was verified by CCK8 assay. (B) Cell invasion and cell migration of SK-N-BE(2) was detected by Transwell assay and wound healing assay. (C) Cell invasion and cell migration of SK-N-SH was detected by Transwell assay and wound healing assay. Scale bar: 100 μm. Scale bar: 100 μm. *P < 0.05, **P < 0.01, ***P < 0.001 compared with the control group. ns, no remarkable difference.

To verify the effect of NR2F6 on NB cell proliferation, we performed cell viability assays using CCK-8 and showed that NR2F6 knockdown inhibited SK-N-BE (2) and SK-N-SH cell proliferation in vitro (Fig 9A). Transwell results and the scratch assay showed that knockdown of NR2F6 expression levels significantly reduced the invasion and migration ability of SK-N-BE (2) and SK-N-SH cells (Fig 9B, 9C).

### NR2F6 may regulate the MAPK signaling pathway

**Fig 10:**
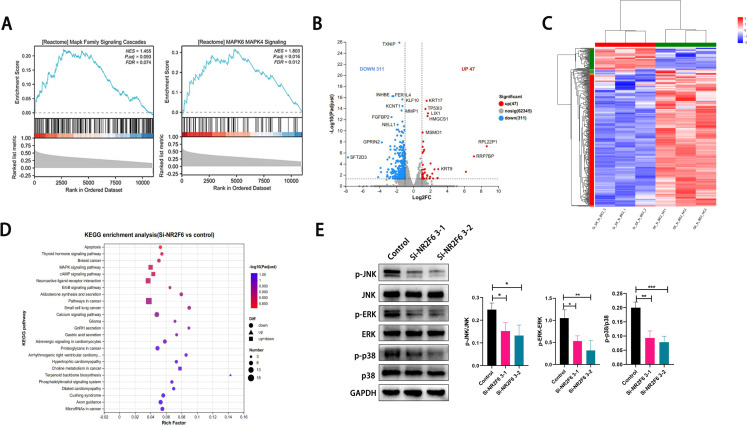
NR2F6 may regulate the MAPK signaling pathway. A:Single-gene GSEA enrichment analysis of the Target-NB data. B:Volcano of differentially expressed genes in NR2F6 down-regulated SK-N-BE (2) and control SK-N-BE (2) cells. C:The cluster analysis heat map of differentially expressed genes in NR2F6 down-regulated SK-N-BE (2) and control SK-N-BE (2) cells. D:KEGG enrichment analysis of differentially expressed genes. E: The expression of the MAPK signaling pathway in SK-N-BE (2) cells before and after NR2F6 downregulation. *P < 0.05, **P < 0.01, ***P < 0.001.

Single-gene GSEA enrichment analysis of the Target-NB data revealed that NR2F6 single-gene GSEA was highly enriched to the MAPK signaling pathway (Fig 10A). To further screen for downstream pathways regulated by NR2F6, we screened for differentially expressed genes in NR2F6 down-regulated SK-N-BE (2) and control SK-N-BE (2) cells by transcriptome sequencing. Volcano results showed that a total of 356 genes were differentially expressed in Si-NR2F6 group and control group, of which 311 genes decreased in Si-NR2F6 group and 47 genes were up-regulated in Si-NR2F6 group (Fig 10B). The cluster analysis heat map indicated significant differences in mRNA between the experimental and control groups (Fig 10C). KEGG enrichment analysis of differential genes showed that differential genes induced by NR2F6 knockdown were also significantly enriched in MAPK signaling pathway, which was consistent with the results of the single gene GSEA enrichment analysis performed by Target-NB data, both highly enriched to MAPK signaling pathway (Fig 10D). So we verified the expression of the MAPK signaling pathway in SK-N-BE (2) cells before and after NR2F6 downregulation. Western blot The results showed that the expression levels of p-JNK, p-ERK and p-p38, key proteins of MAPK signaling pathway, were significantly inhibited after the downregulation of NR2F6 expression (Fig 10E). The above results suggest that NR2F6 gene may regulate the expression of MAPK signaling pathway to regulate downstream biological behavior.

## Discussion

Cancer-related research is currently a prominent focus in medicine. Given that cancer arises from a combination of abnormal cell proliferation, differentiation, and immune stress, identifying genes that can concurrently impact these processes for targeted therapies has become a compelling avenue of investigation. In our literature search, we discovered that NR2F6 exhibits dual tumor-promoting activities in both the immune system and tumor cells. Nevertheless, the role of NR2F6 in pancancer remains poorly understood. To address this gap, we conducted gene expression difference analysis using data from 34 cancer-related sources in the UCSC database, revealing a significant up-regulation of NR2F6 in various cancers. Our study thoroughly explored NR2F6 expression in cancer and normal tissues, highlighting significant differences and discussing its potential as a predictive marker.

NR2F6 (Ear-2) is an orphan nuclear receptor with varying expression levels in different standard and tumor tissues. Our study demonstrated that NR2F6 was differentially expressed in most tumors and adjacent normal tissues. Analysis of mRNA expression profiles and protein expression levels revealed significant up-regulation in 25 cancer tissues and significant down-regulation in low-grade glioma (LGG), papillary cell carcinoma of the kidney (KIPAN), and clear cell carcinoma of the kidney (KIRC) tissues, suggesting distinct roles for NR2F6 in the development of different cancers. Furthermore, we evaluated the relationship between NR2F6 expression levels and OS, DSS, DFI, and PFI, finding correlations with the prognosis of various cancers. Previous studies have also shown increased NR2F6 expression in leukemia patients [10], breast cancer with poor prognosis [25], cervical cancer with pelvic lymph node metastasis and poor prognosis [7], and chemotherapy-resistant ovarian cancer [5]. Additionally, NR2F6 has been associated with colorectal cancer progression and hepatocellular carcinoma cell proliferation and metastasis [2,26]. Together with our pancancer analysis, these results suggest that NR2F6 may serve as a potential marker for poor prognosis in different cancers, further indicating its involvement in cancer progression.

NB is a cancer originating from neural crest cells and can develop anywhere in the sympathetic nervous system. It is among the most common malignant solid tumors in childhood and infancy, accounting for approximately 8% of childhood cancers and 15% of childhood cancer-related deaths [27]. NB is a complex disease, exhibiting biological, clinical, morphological, and genetic heterogeneity. Despite modified multimodal therapies, nearly half of high-risk patients do not respond to initial treatments or experience recurrence within the first two years of treatment [28]. The prognosis for high-risk children is grim, with a 5-year survival rate of less than 50% [12]. The variable response of children to standard therapy is largely attributed to multidrug resistance and drug toxicity [29,30]. NB has thus become a significant health problem and a clinical treatment challenge, seriously impacting children’s well-being. Consequently, finding specific therapeutic targets for NB and improving children’s survival rate has become an urgent research priority. In light of this, we focused on investigating the role of the orphan nuclear receptor NR2F6 in retroperitoneal NB, validating its potential as a prognostic factor and its significance in cancer development. According to the pancancer analysis, we found that the overall survival of children in the NR2F6 high expression group was worse than that of the NR2F6 low expression group; in addition, multivariate Cox regression analysis confirmed that NR2F6 expression level, COG risk and MKI were independent risk factors affecting OS in NB children, emphasizing the importance of NR2F6 for the evaluation of prognosis of NB children. In addition, we examined the NR2F6 expression level in NB tumor samples from our hospital and divided them into two groups of high and low expression. A multivariate Cox regression analysis combined with clinicopathological information also confirmed that NR2F6 expression level was closely correlated with OS in children with NB. In addition, the analysis of NB clinical data in our hospital also observed a statistical difference in the organ metastasis index of the high and low NR2F6 expression group, and organ metastasis was a risk factor for OS in children with NB. All the above results suggest that NR2F6 expression level may be closely related to NB progression and metastatic recurrence.

NR2F6 regulates cell growth at specific stages of differentiation, with overexpression inhibiting granulocyte and monocyte differentiation, while under-expression induces granulocyte differentiation [10]. Our immunohistochemistry results aligned with this finding, as poorly differentiated tumor cells exhibited high NR2F6 expression, with a shift from nuclear to cell membrane localization, showing nuclear membrane-positive expression. In nodular cell NB, NR2F6 expression was low, primarily concentrated in the nucleus, with significantly reduced expression in the cell membrane. This raises the question of whether nuclear membrane migration of NR2F6 is indicative of tumor progression or related to the cell differentiation mechanism, necessitating further experimental exploration.

NR2F6 is a transcription factor that can influence the tumor microenvironment in various ways. In the inflammatory tumor microenvironment, NR2F6 is highly inducible in effector T cells, locally activating T-cell immunity and reducing immune-related adverse effects [31]. NR2F6 in T-cells inhibits the transcription of the cytokines NFAT and AP-1, which suppresses activated secretion of IL-2 IFNγ and IL17 in activated T cells, promoting an aberrant immune response to tumors [32]. NR2F6 is commonly expressed in resting T cells at low levels; however, it is highly inducible in effector (but not regulatory) T cells in the inflammatory tumor microenvironment [33]. In vitro studies have shown that NR2F6 is an inhibitory receptor in tumor-associated non-regulatory T cells, highly expressed on so-called “exhausted” T cells to reactivate tumor-specific T lymphocytes [34]. Upregulation produces effector T cells that are unable to achieve an adequate immune response to cancer, and NR2F6 downregulation sensitizes tumors to established PD-1/PD-L1 axis blockade, which produces a potent synergistic effect with established surface checkpoint blockade (PD-L1, CTLA-4) to inhibit tumor progression [30]. Ablation of NR2F6 enhances the expression of interleukin-2 (IL-2), interferon -γ (IFN-γ), and tumor necrosis factor-α (TNF-α) secretion, thereby promoting anti-tumor immune responses. NR2F6 gene ablation/PTT-mediated aPD-L1 immune-enhancing approach allows local treatment-stimulated immune responses to build long-term immune memories throughout the body, thereby inhibiting tumor metastasis [8]. Immune cell infiltration analysis based on TIMER algorithm found that the expression of NR2F6 was significantly negatively associated with T cell infiltration in most cancers, while that the high NR2F6 expression was associated with worse prognosis, suggesting that targeted inhibition of NR2F6 expression level in pancancer may induce increased T cell infiltration, thus enabling tumor benefit in immunotherapy. Moreover, in NB, based on the CIBERSORT algorithm, we observed that NR2F6 expression was inversely associated with immune cell infiltration such as T_cells_CD4_memory_activated, Master _ cell _ activated, Neutrophils. This result suggests that targeted inhibition of NR2F6 expression in NB may induce increased infiltration of these immune cells to make NB benefit in immunotherapy.

Through the results of pancancer analysis and clinical data, we found that NR2F6 was significantly related to NB prognosis and metastasis, suggesting that targeting NR2F6 may be one of the potential strategies for NB treatment. We further explored the role of NR2F6 in NB at the cellular level. Review of the clinicopathological analysis of the NB data in our hospital found that the MYCN amplification status between the high and low NR2F6 expression groups was significantly different. Therefore, we also performed relevant validation at the NB cell level, and we found that the NR2F6 expression level in MYCN-amplified NB cells was significantly higher than that in MYCN non-amplified NB cells. Subsequently, we downregulated NR2F6 expression levels in both MYCN-amplified NB cells and MYCN-non-amplified NB cells and performed related functional experiments. The results found that downregulation of NR2F6 expression inhibited NB proliferation, migration and invasion in both cells. The above findings suggest that targeted inhibition of NR2F6 expression can indeed alter the malignant biological behavior of NB, making NR2F6 one of the potential targets for NB therapy. To explore the downstream pathways regulated by NR2F6, we further performed a single-gene GSEA analysis using the target-NB data, which was found to be highly enriched in the MAPK signaling pathway. Subsequently, NB cells before and after NR2F6 down-regulation were used for RNA sequencing, and the gene changes induced by NR2F6 downregulation were screened, and the differential genes were also highly enriched in MAPK related pathways. Further, the MAPK signaling changes induced by NR2F6 downregulation were detected by western blot, and it was found that NR2F6 downregulation inhibited the expression level of key proteins of MAPK signaling pathway. Previous studies have found that the MAPK pathway is active in the medium tumor microenvironment of macrophage/ microglia, which is involved in microglia/ macrophage polarization, etc [35]. In addition, several studies have shown that dysregulation of the tumor microenvironment and immune cell activity in some tumor entities may lead to resistance to MAPK pathway inhibitors [36,37]. These studies suggest that the MAPK pathway may also be involved in immune regulation in the tumor microenvironment. Pancancer analysis suggests that the expression level of NR2F6 is correlated with the level of certain immune cell infiltration in NB, but whether the MAPK pathway induced dysregulation of immune cell activity or altered immune cell infiltration level in NB still needs further exploration.

## Conclusion

In this study, the pancancer analysis of NR2F6 found that NR2F6 appeared as a tumor-promoting factor in various tumor types, and its expression was closely associated with patient prognosis, and higher expression was associated with reduced survival time. Moreover, NR2F6 plays a unique role in the tumor immune microenvironment, affecting the immune infiltration within the tumor. We focused on the expression level and role of NR2F6 in NB. We found that NR2F6 was an independent risk factor for the prognosis of children with NB, and in vitro cell experiments confirmed that NR2F6 downregulation may reduce the malignant biological behavior of NB cells. These common findings make NR2F6 a promising biomarker and potential therapeutic target for the prediction of NB prognosis. However, the mechanism by which NR2F6 regulates NB progression is still unclear, and whether the altered level of immune cell infiltration plays a role in the therapeutic effects of targeted inhibition of NR2F6 on NB still needs further exploration.

## Supporting information

S1 Images S1 raw images(1). (PDF)

S1 Table NR2F6 siRNA sequence. (PDF)

S2 Table Sequence-specific primers. (PDF)

S3 Table Antibodies Used for Different Experiments. (PDF)

S4 Table Abbreviations and full name of pan-cancers. (PDF)

S5 Table Gene expression data related to the prognosis of children with neuroblastoma in the TARGET database. Transcriptomic profiles and matched clinical records were acquired from the Therapeutic Applicable Research to Generate Effective Treatments (TARGET) database. To ensure data quality, we implemented a stringent filtering criterion: transcripts with expression counts below 3 in ≥10% of samples were excluded from subsequent analysis. For survival outcome assessment, univariate Cox proportional hazards regression modeling was performed using the survival package (v3.4.0) in R, incorporating both temporal survival parameters and overall survival (OS) endpoints. Statistical significance was determined at a predefined threshold of P < 0.05 (two-tailed).
(CSV)
