# Supplementary material for: NR2F6 promotes the malignant progression of neuroblastoma as an indicator of poor prognosis
Source: PLoS One. 2025 May 27;20(5):e0324334. doi: 10.1371/journal.pone.0324334 (PMC12112146; doi:10.1371/journal.pone.0324334)
Supplement: S1 Images — (PDF) [file pone.0324334.s001.pdf]

Figure8A: The order of addition of samples was respectively: SK-N-DZ、SK-N-BE（2）、SK-N-SH, all Western blot experiments were repeated 3 times. The red boxes mark the results as bands in the manuscript.

NR2F6（43KDa）

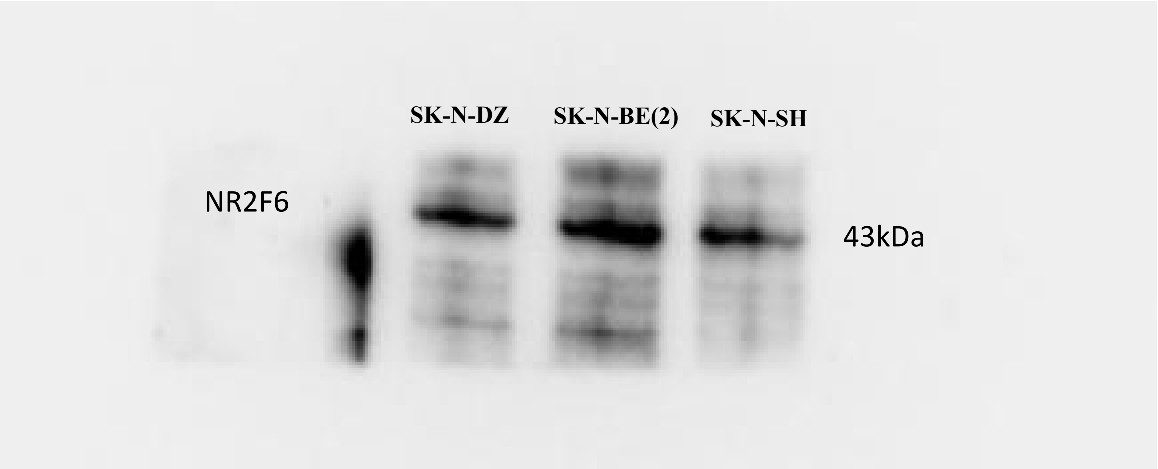

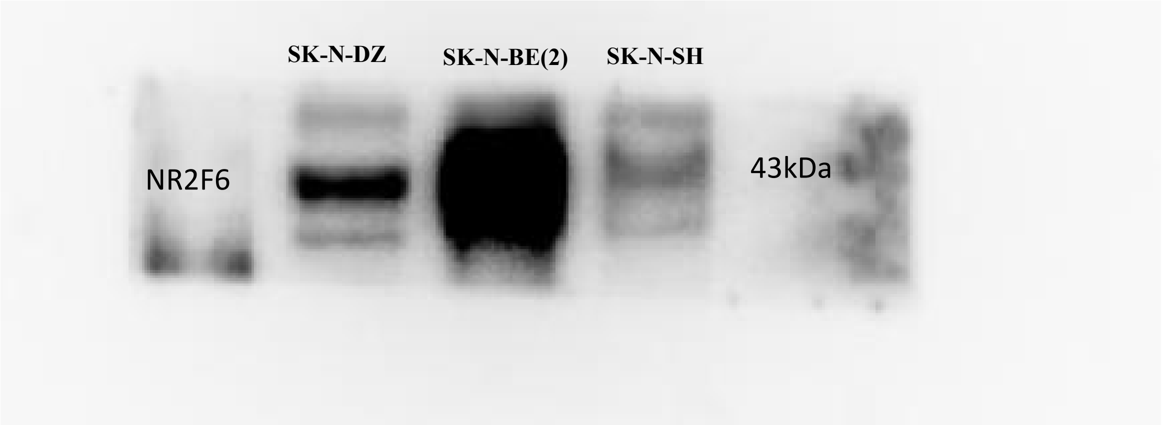

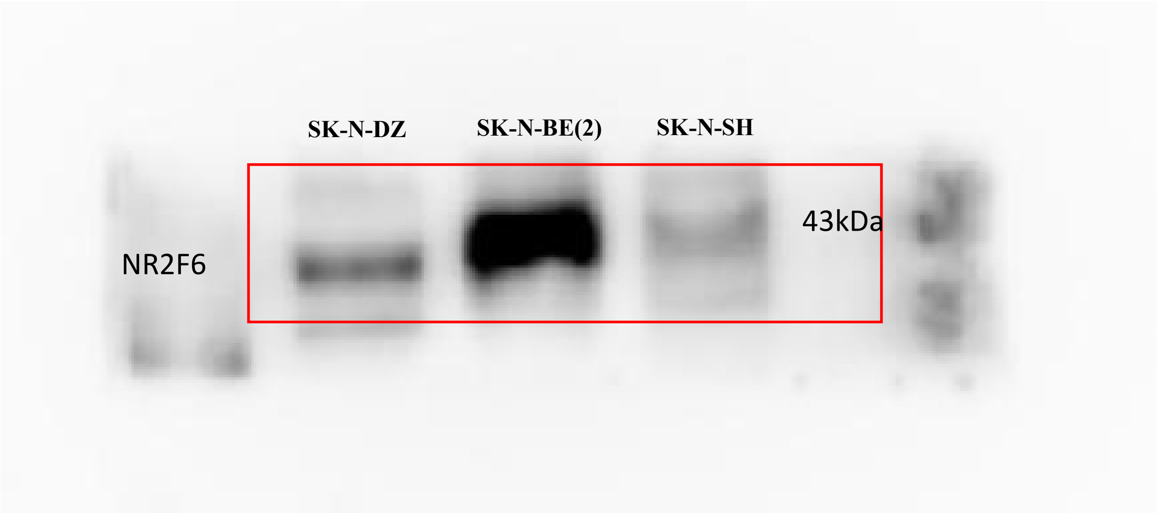

GAPDH（36KDa）

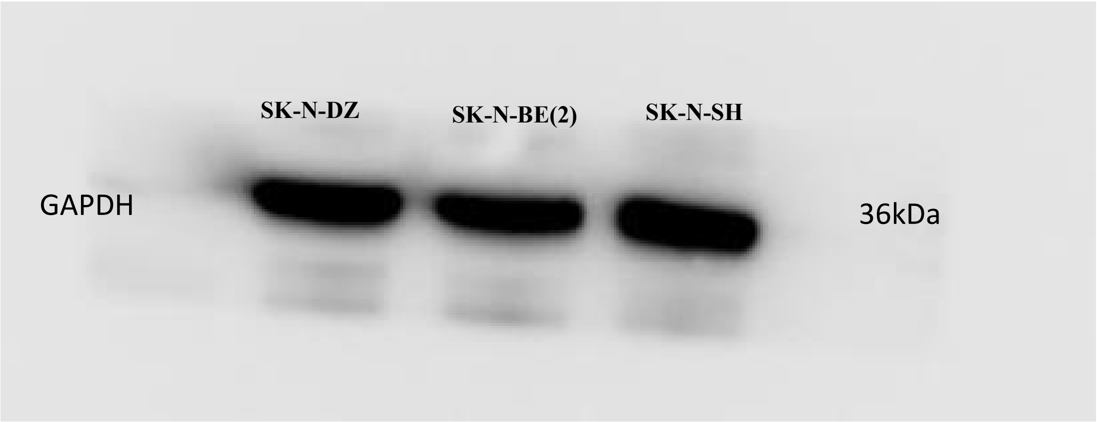

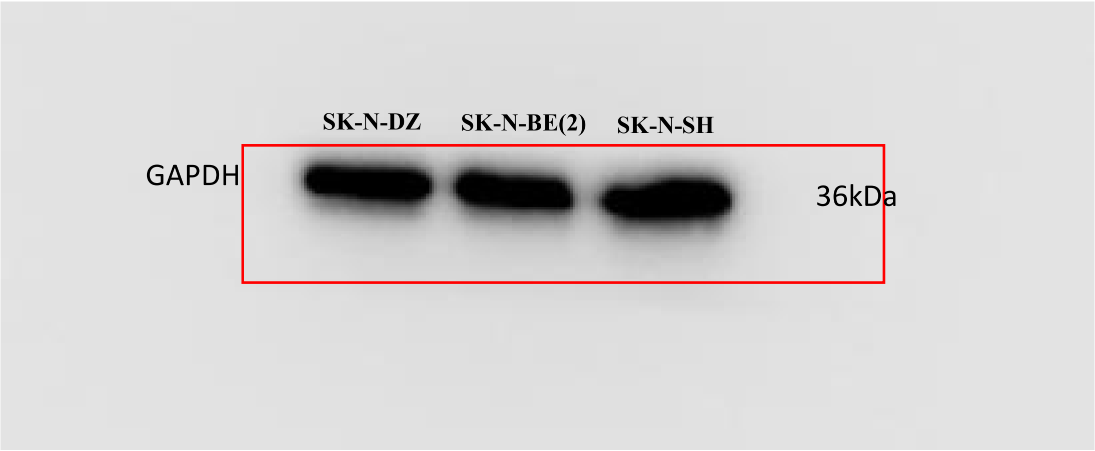

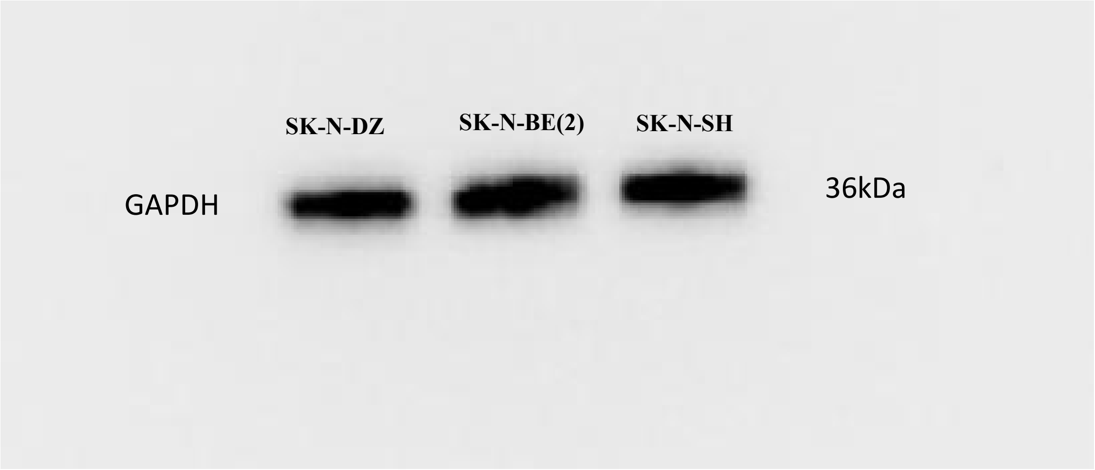

Figure8D: The order of addition of samples was respectively: Control、SiNR2F6 3-1、SiNR2F6 3-2, all Western blot experiments were repeated 3 times. The red boxes mark the results as bands in the manuscript.

SK-N-BE（2）（43KDa）

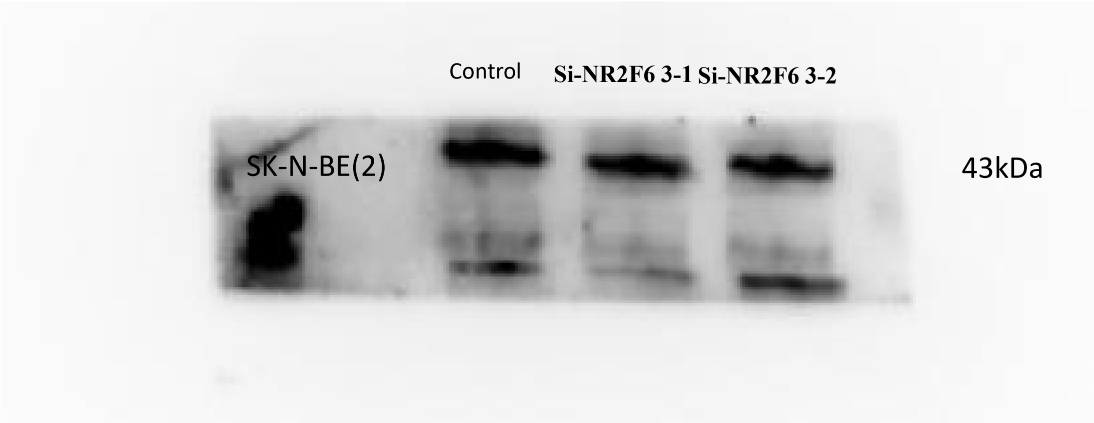
Control
Si-NR2F6 3-1
Si-NR2F6 3-2
SK-N-BE(2)
43kDa

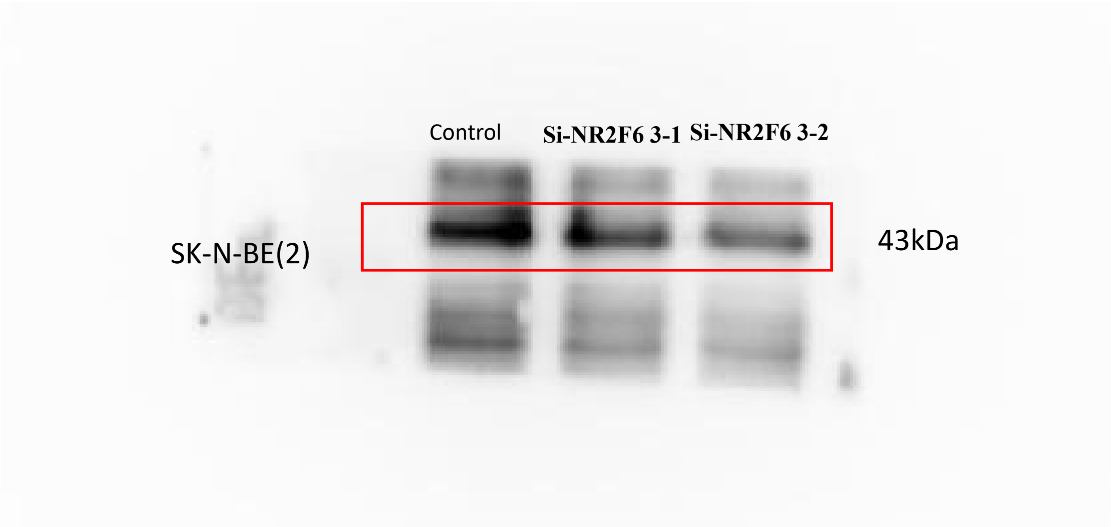
Control
Si-NR2F6 3-1
Si-NR2F6 3-2
SK-N-BE(2)
43kDa

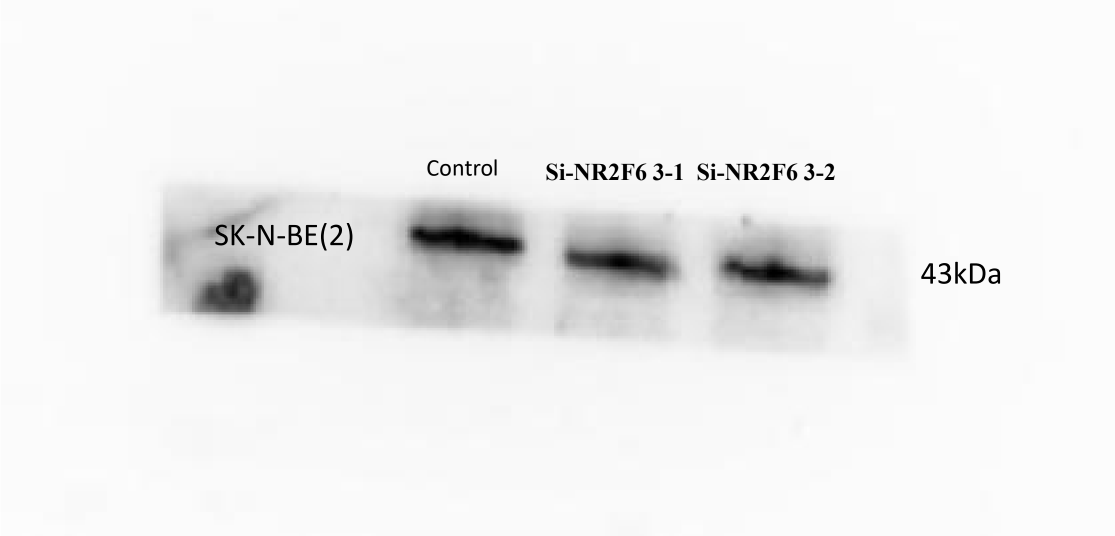
Control
Si-NR2F6 3-1
Si-NR2F6 3-2
SK-N-BE(2)
43kDa

GAPDH（36KDa）

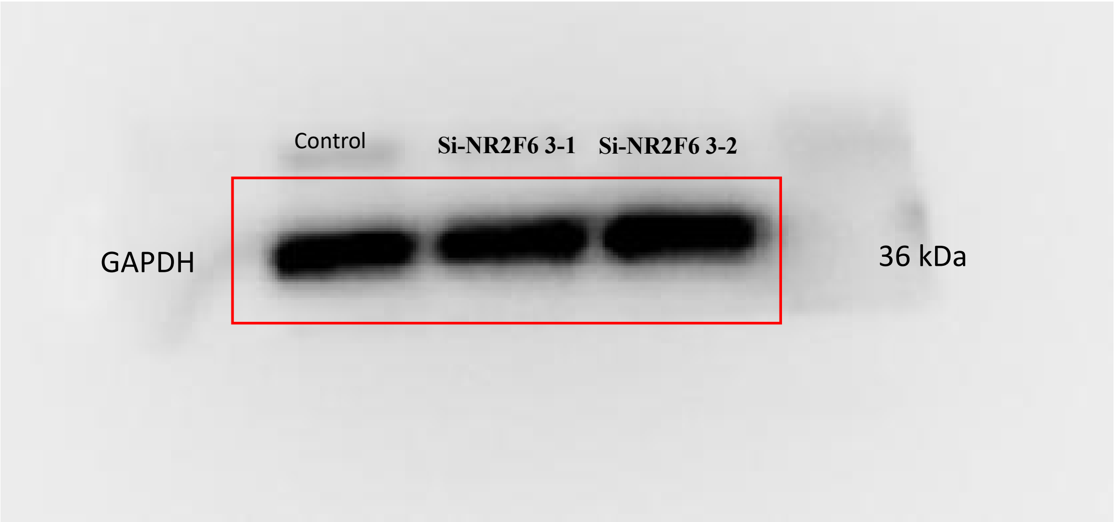
Control
Si-NR2F6 3-1
Si-NR2F6 3-2
GAPDH
36 kDa

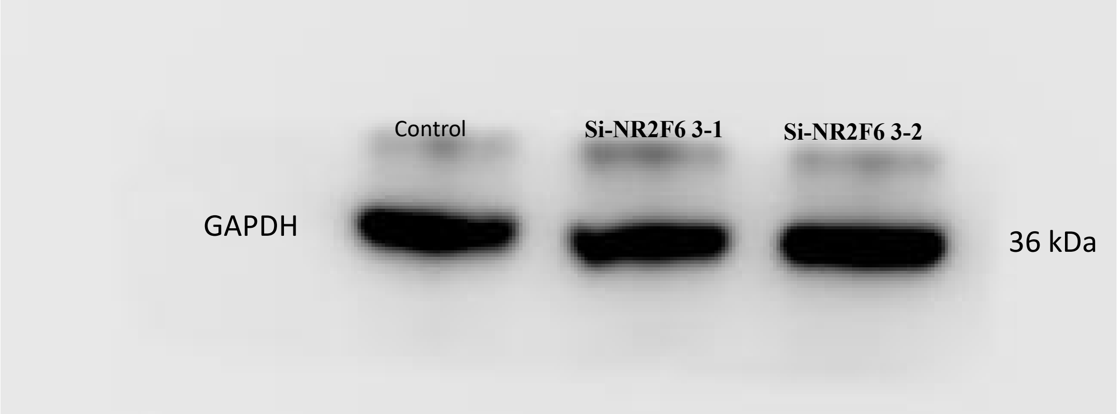
Control
Si-NR2F6 3-1
Si-NR2F6 3-2
GAPDH
36 kDa

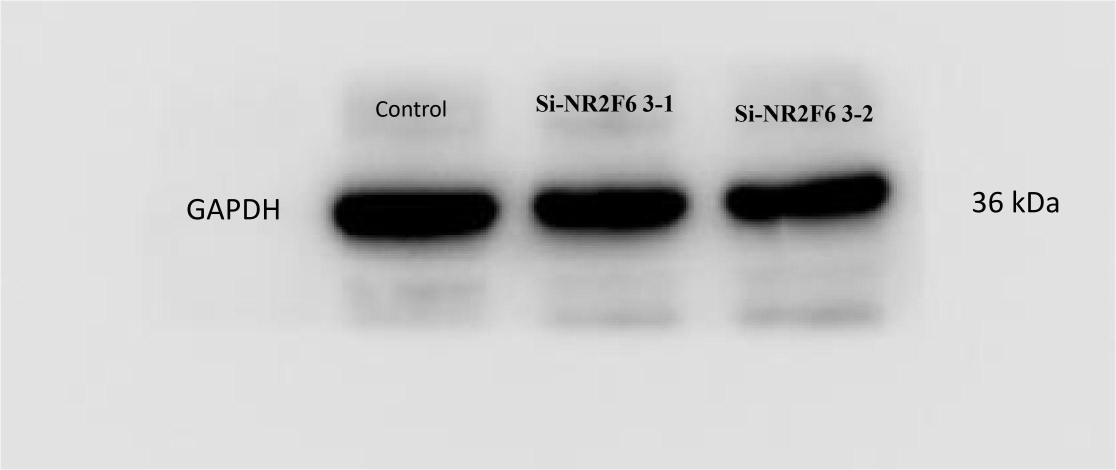
Control
Si-NR2F6 3-1
Si-NR2F6 3-2
GAPDH
36 kDa

SK-N-SH（43KDa）

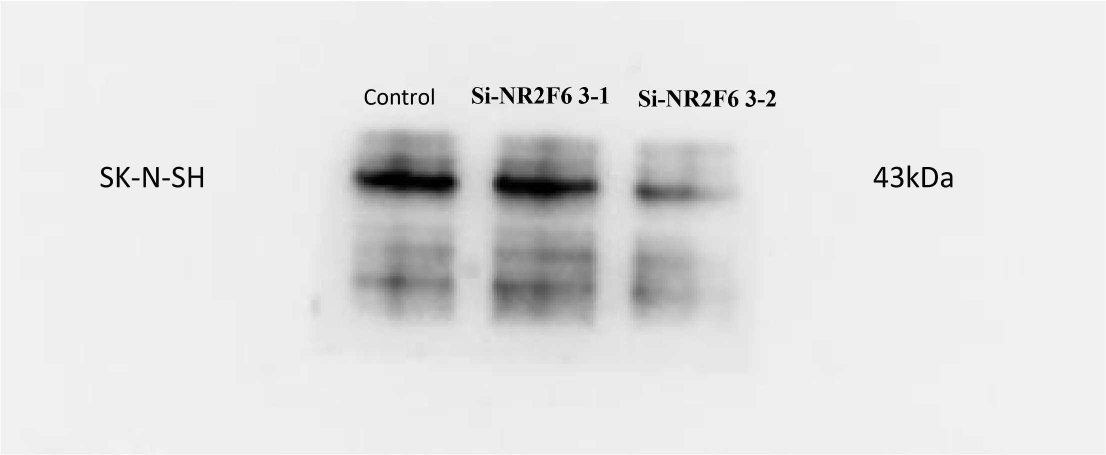
Control
Si-NR2F6 3-1
Si-NR2F6 3-2
SK-N-SH
43kDa

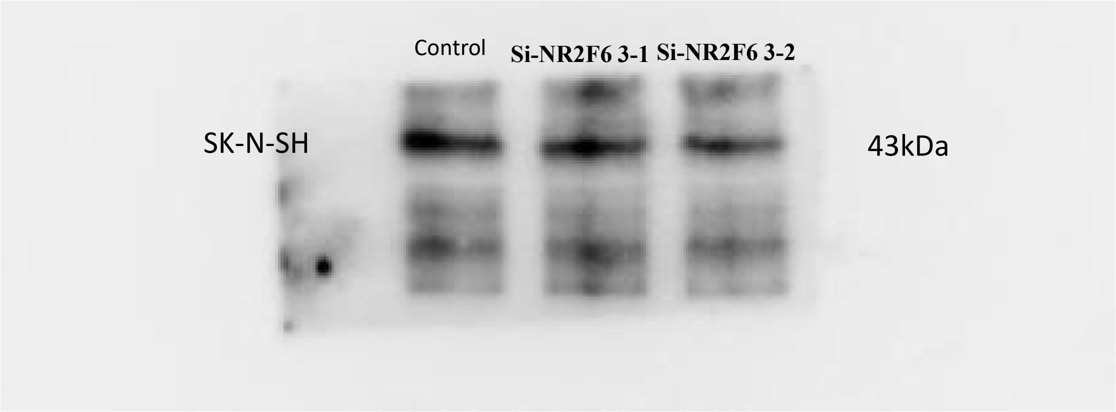
Control
Si-NR2F6 3-1
Si-NR2F6 3-2
SK-N-SH
43kDa

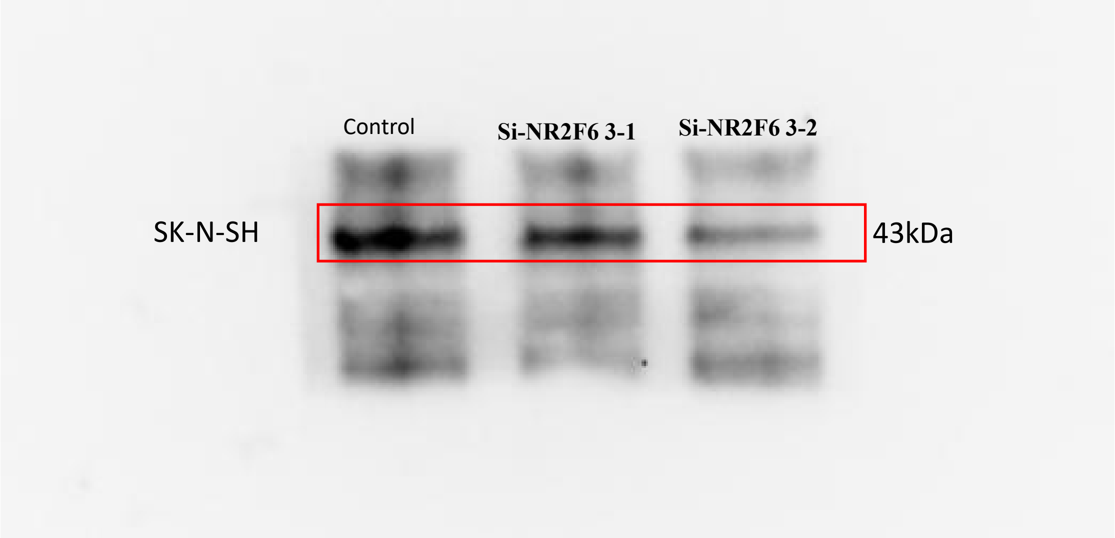
Control
Si-NR2F6 3-1
Si-NR2F6 3-2
SK-N-SH
43kDa

GAPDH（36KDa)

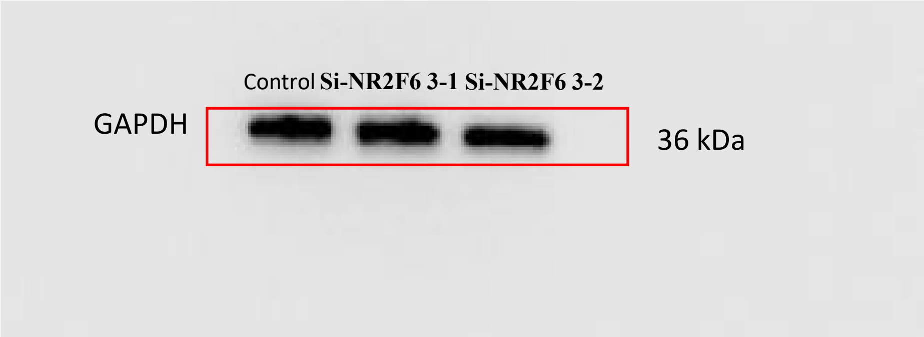
Control
Si-NR2F6 3-1
Si-NR2F6 3-2
GAPDH
36 kDa

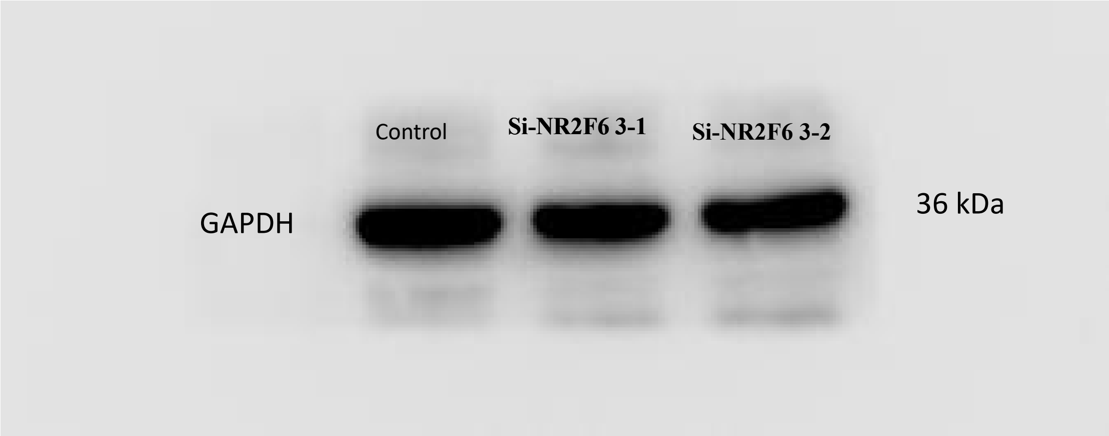

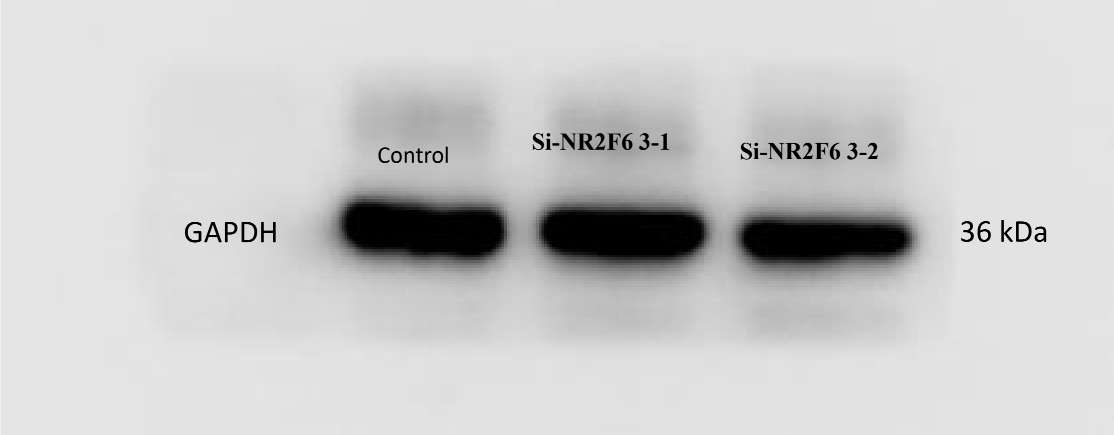

Figure10E: The order of addition of samples was respectively: Control、SiNR2F6 3-1、SiNR2F6 3-2, all Western blot experiments were repeated 3 times. The red boxes mark the results as bands in the manuscript.

p-JNK (48KDa）

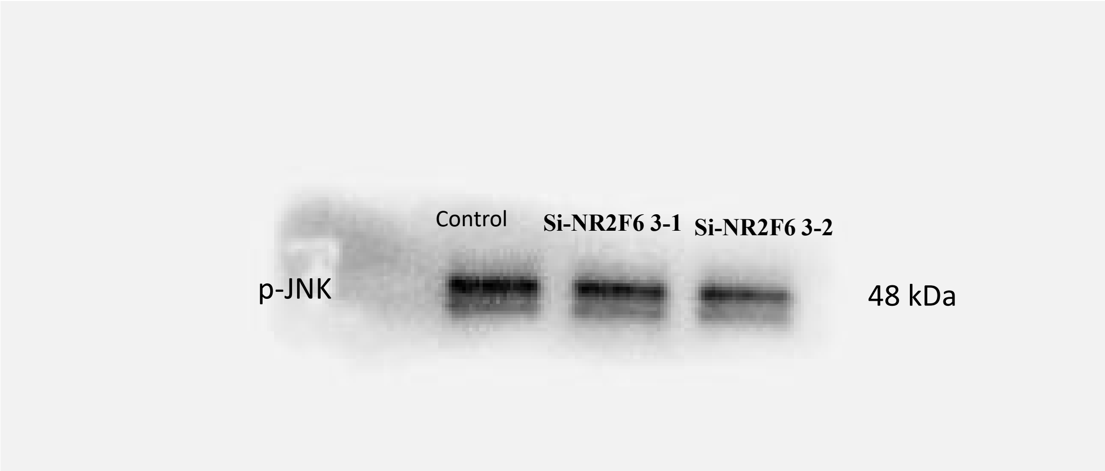

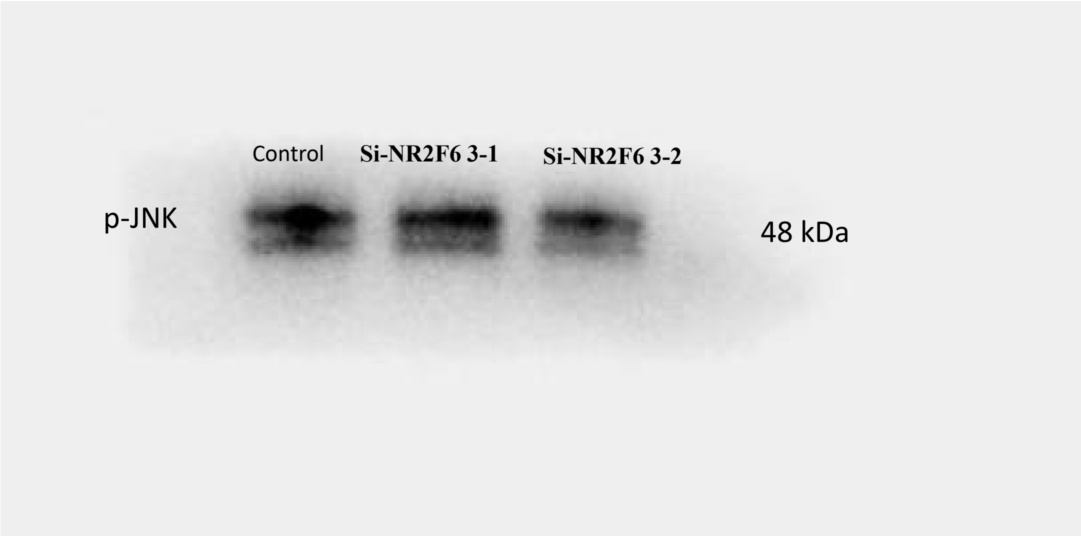
Control
Si-NR2F6 3-1
Si-NR2F6 3-2
p-JNK
48 kDa

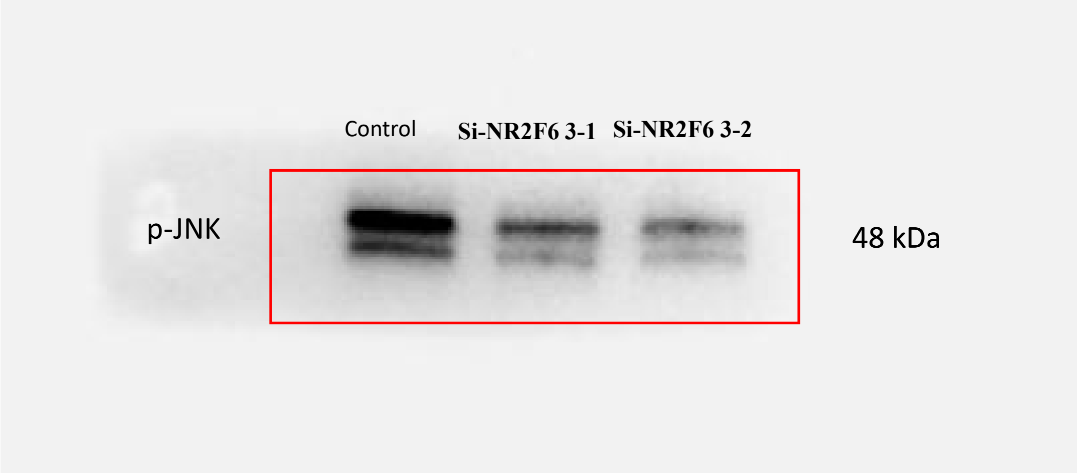
Control
Si-NR2F6 3-1
Si-NR2F6 3-2
p-JNK
48 kDa

p-P38（43KDa）

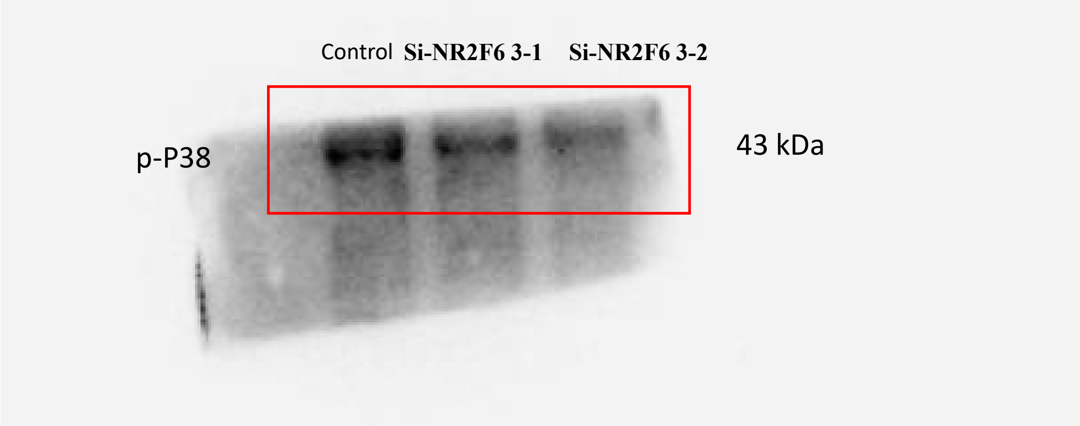
Control
Si-NR2F6 3-1
Si-NR2F6 3-2
p-P38
43 kDa

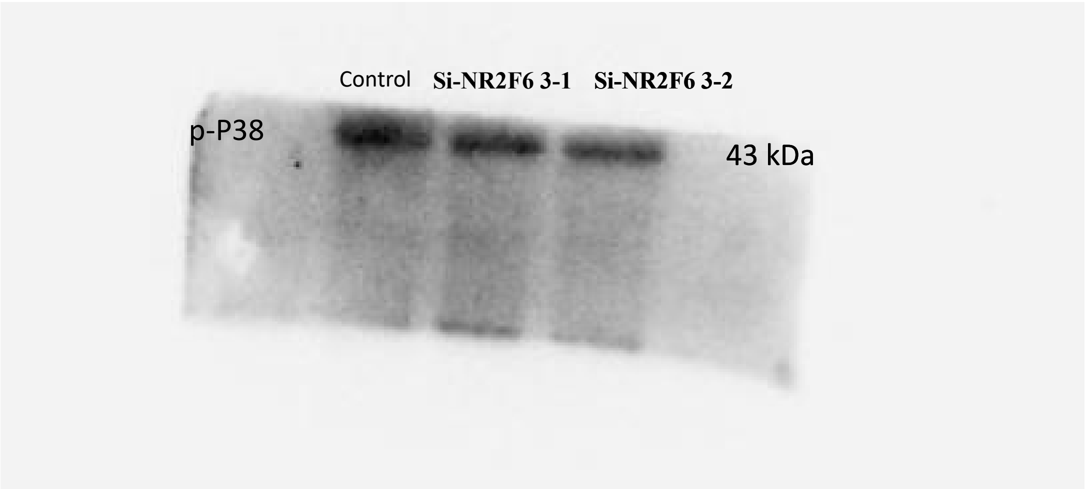
Control
Si-NR2F6 3-1
Si-NR2F6 3-2
p-P38
43 kDa

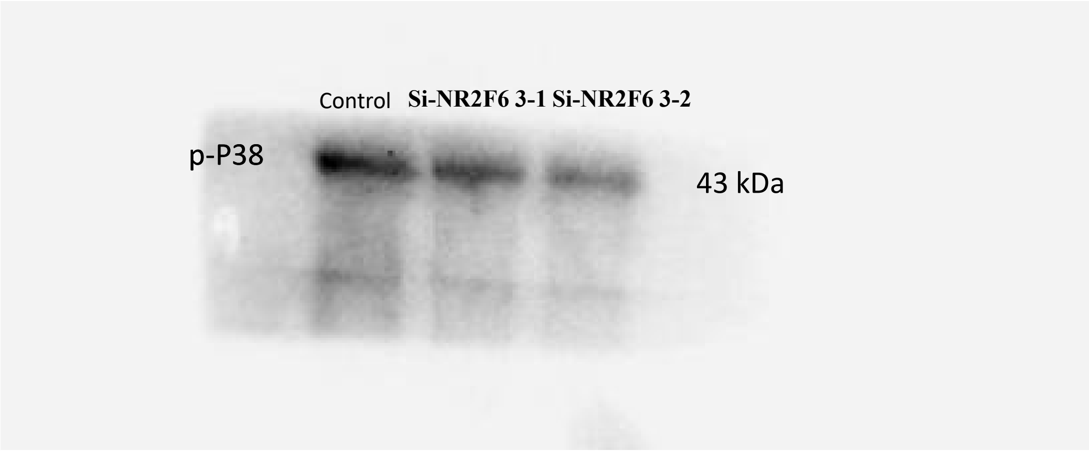
Control
Si-NR2F6 3-1
Si-NR2F6 3-2
p-P38
43 kDa

GAPDH（36KDa）

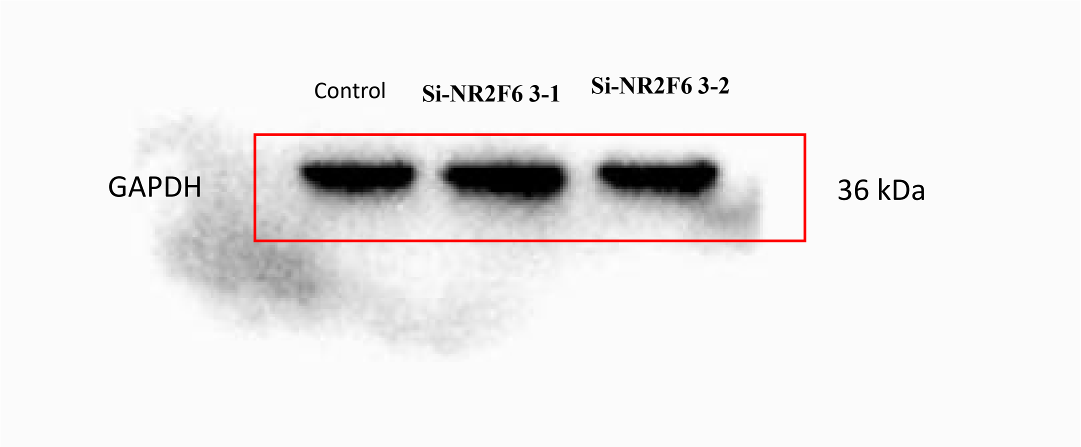
Control
Si-NR2F6 3-1
Si-NR2F6 3-2
GAPDH
36 kDa

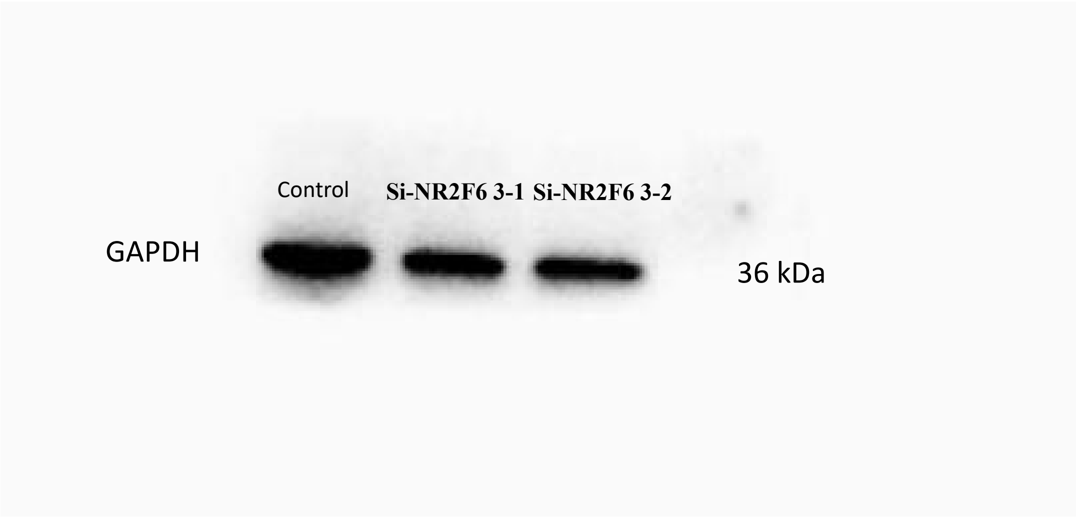
Control
Si-NR2F6 3-1
Si-NR2F6 3-2
GAPDH
36 kDa

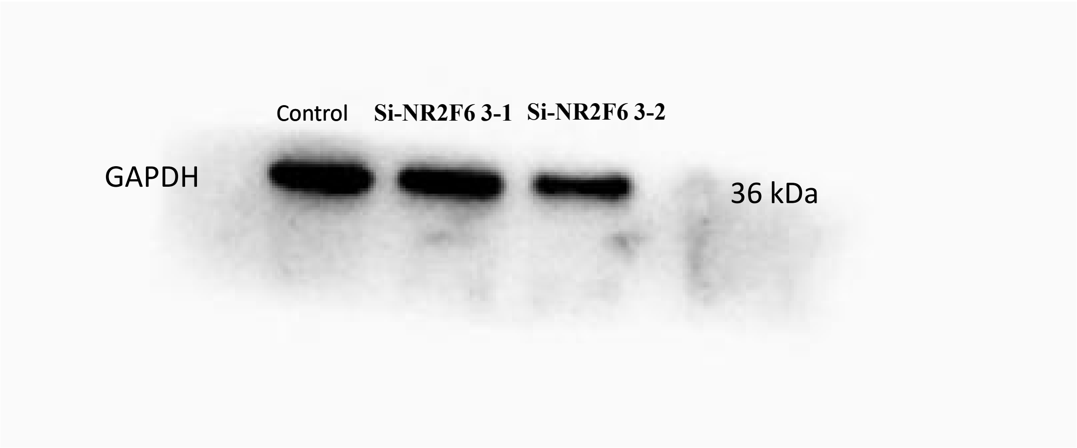
Control
Si-NR2F6 3-1
Si-NR2F6 3-2
GAPDH
36 kDa

JNK（48KDa）

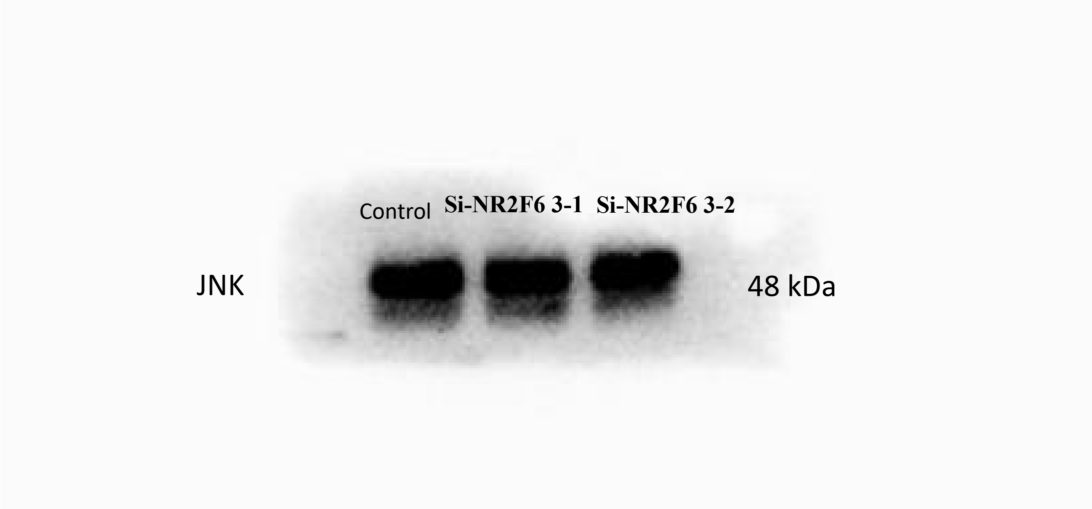
Control
Si-NR2F6 3-1
Si-NR2F6 3-2
JNK
48 kDa

Control

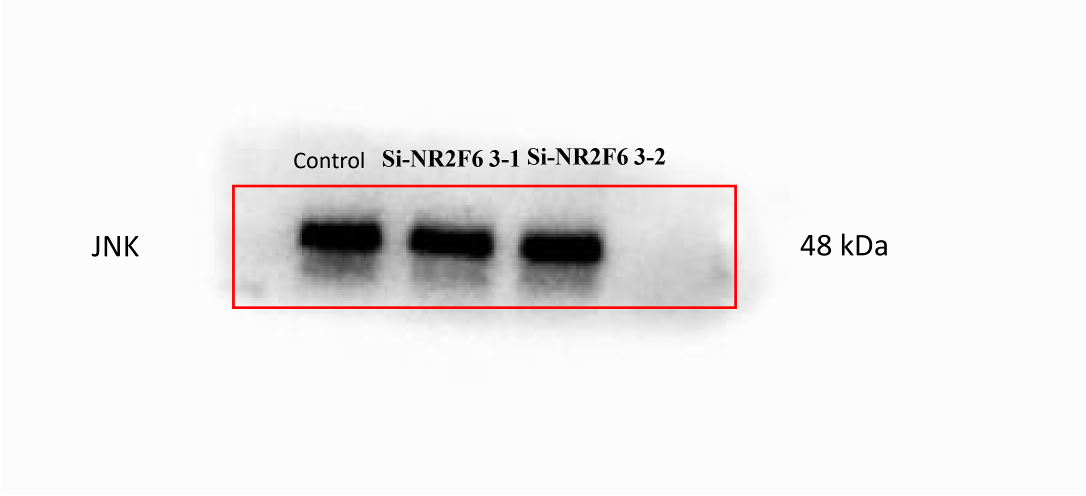

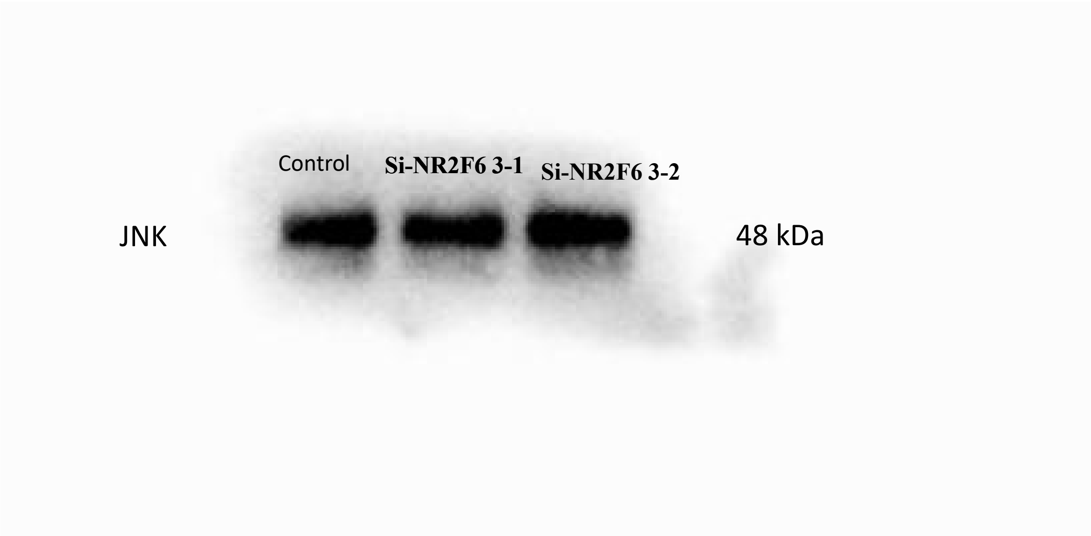

P38（43KDa)

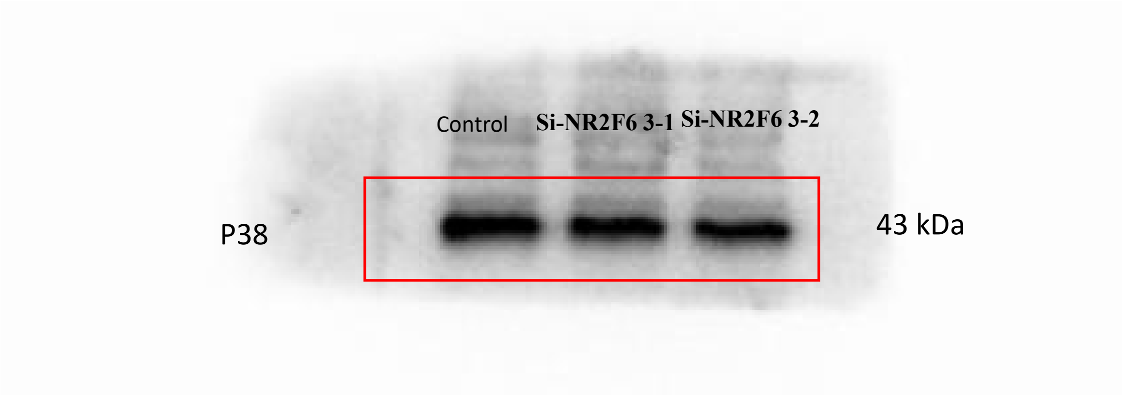

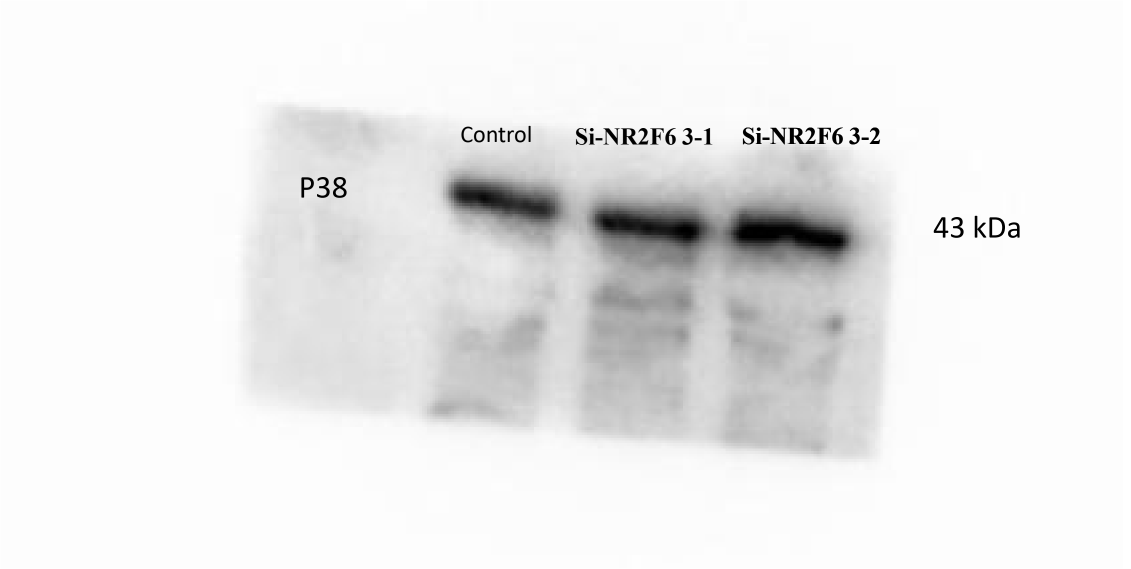
Control
Si-NR2F6 3-1
Si-NR2F6 3-2
P38
43 kDa

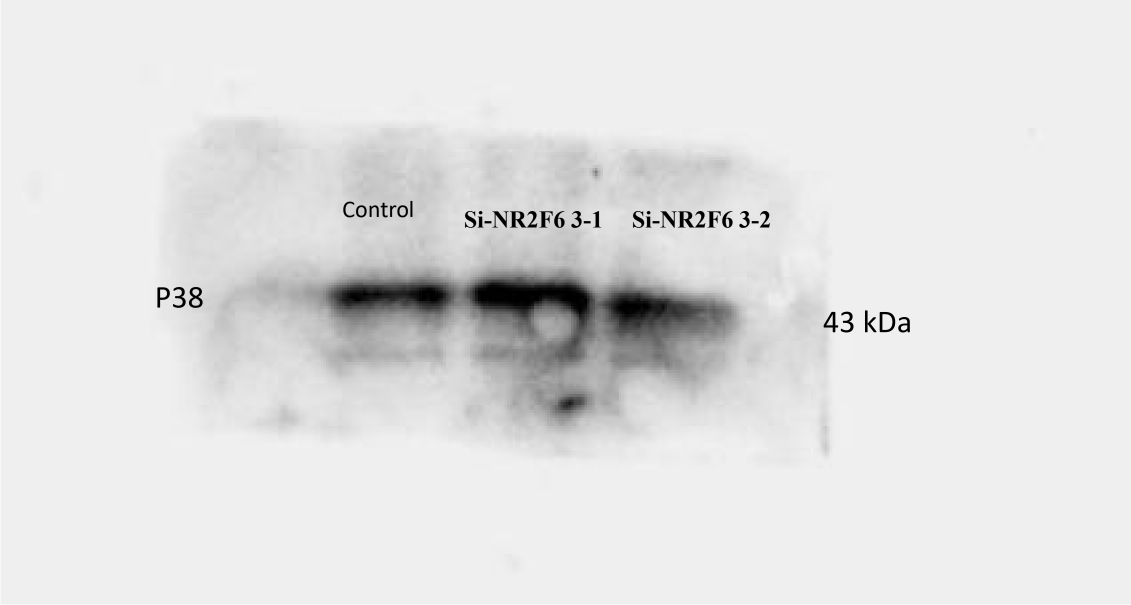
Control
Si-NR2F6 3-1
Si-NR2F6 3-2
P38
43 kDa

GAPDH (36KDa)

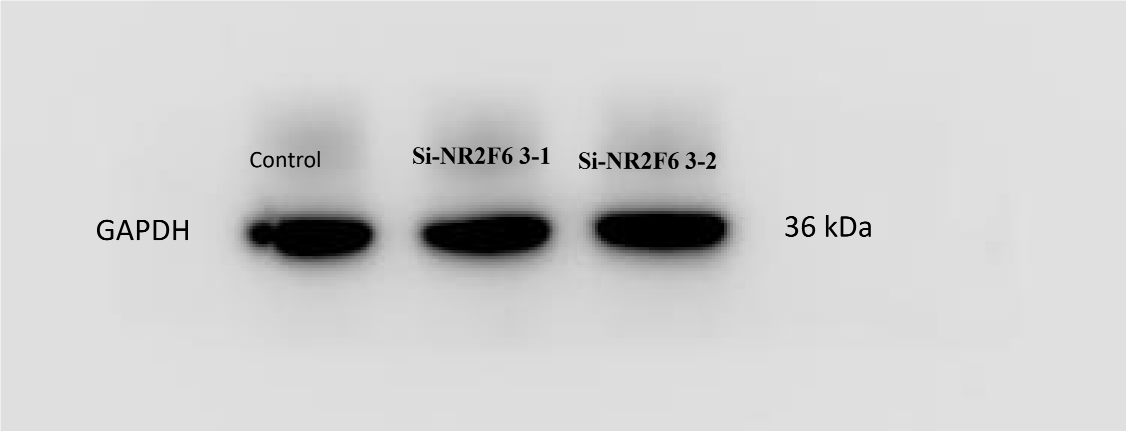
Control
Si-NR2F6 3-1
Si-NR2F6 3-2
GAPDH
36 kDa

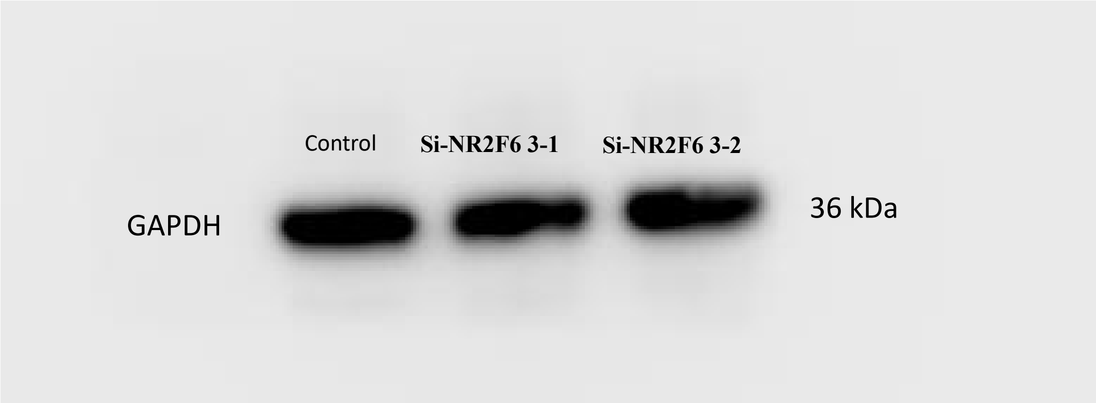
Control
Si-NR2F6 3-1
Si-NR2F6 3-2
GAPDH
36 kDa

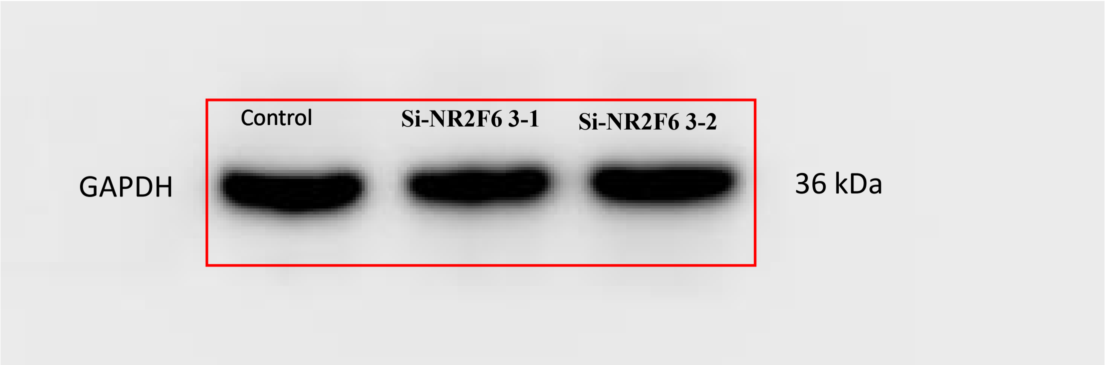
Control
Si-NR2F6 3-1
Si-NR2F6 3-2
GAPDH
36 kDa

P-ERK（43KDa)

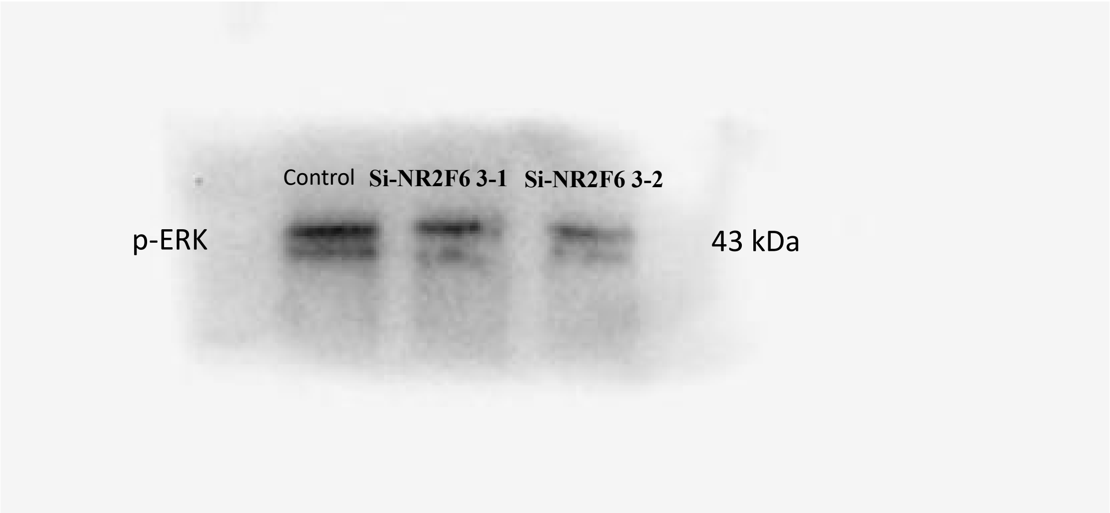
Control
Si-NR2F6 3-1
Si-NR2F6 3-2
p-ERK
43 kDa

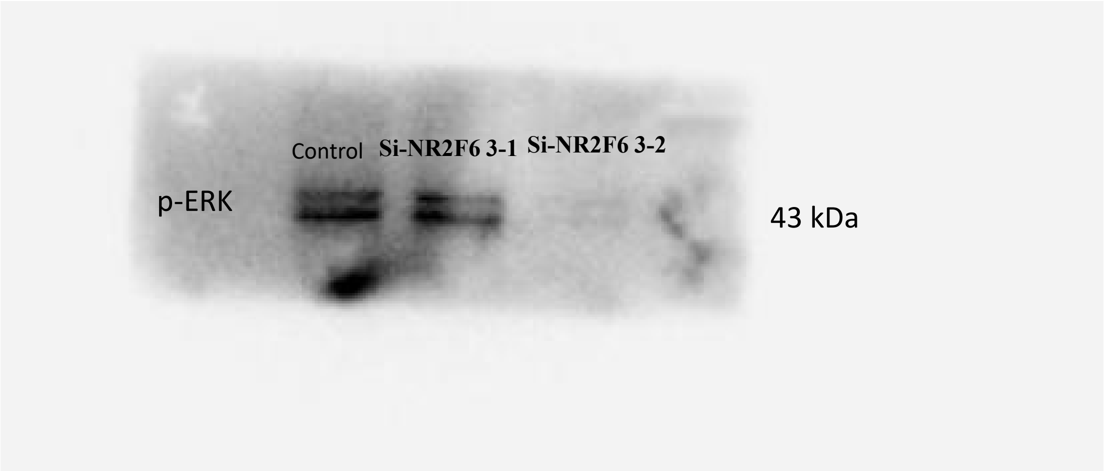
Control
Si-NR2F6 3-1
Si-NR2F6 3-2
p-ERK
43 kDa

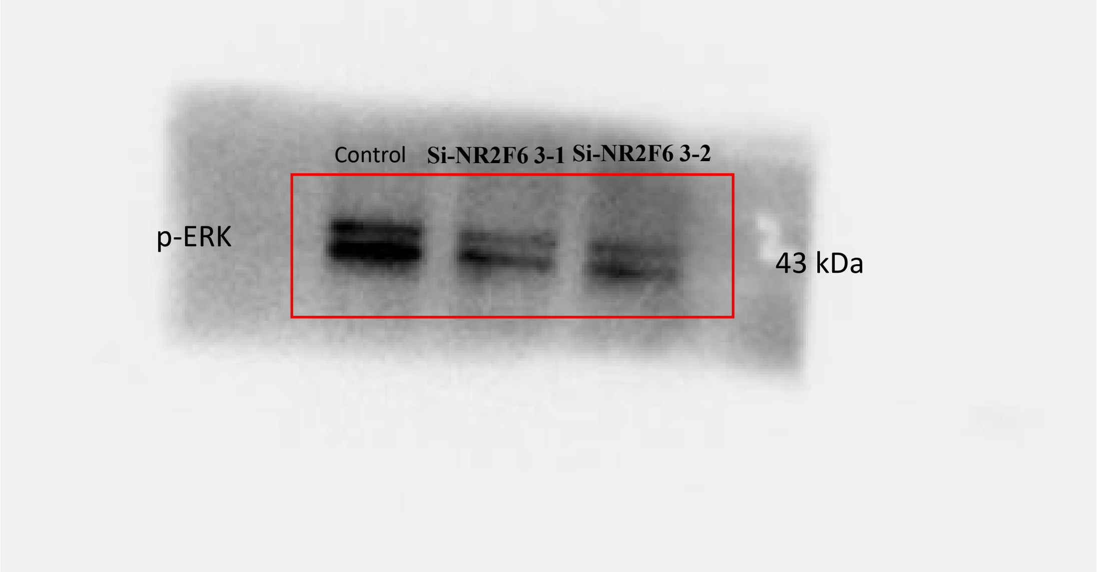
Control
Si-NR2F6 3-1
Si-NR2F6 3-2
p-ERK
43 kDa

GAPDH (36KDa)

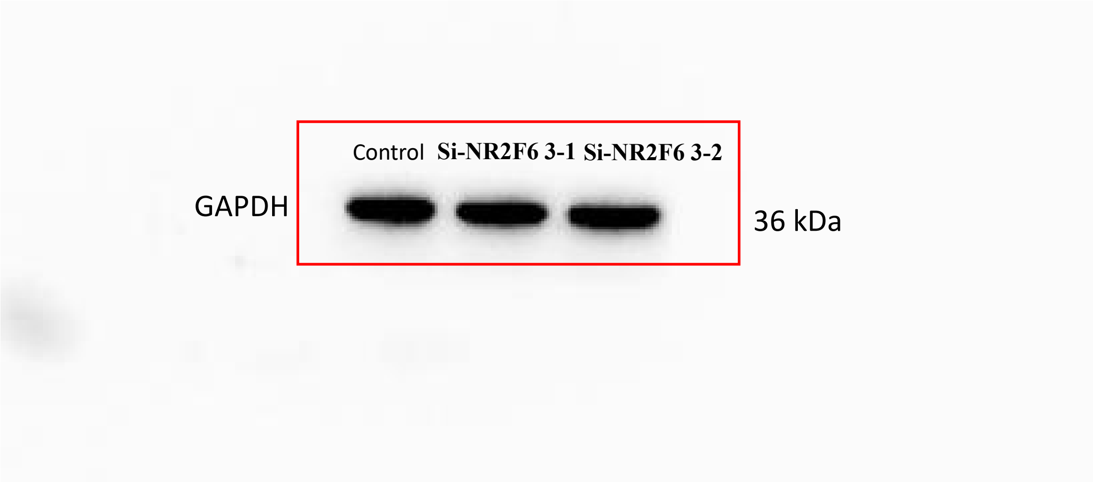
Control
Si-NR2F6 3-1
Si-NR2F6 3-2
GAPDH
36 kDa

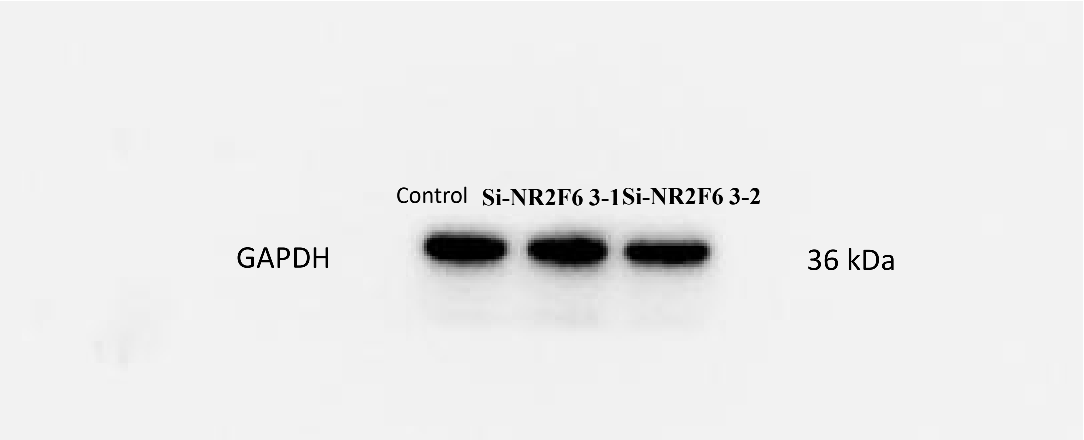
Control Si-NR2F6 3-1Si-NR2F6 3-2
GAPDH
36 kDa

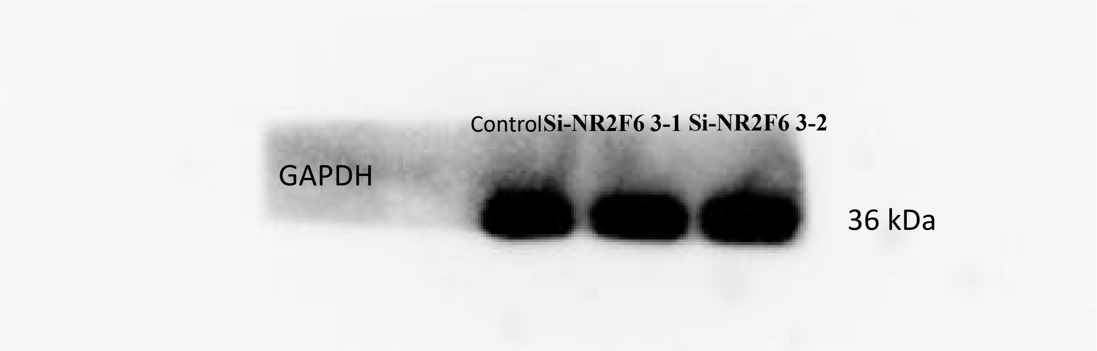
ControlSi-NR2F6 3-1 Si-NR2F6 3-2
GAPDH
36 kDa

ERK（43KDa)

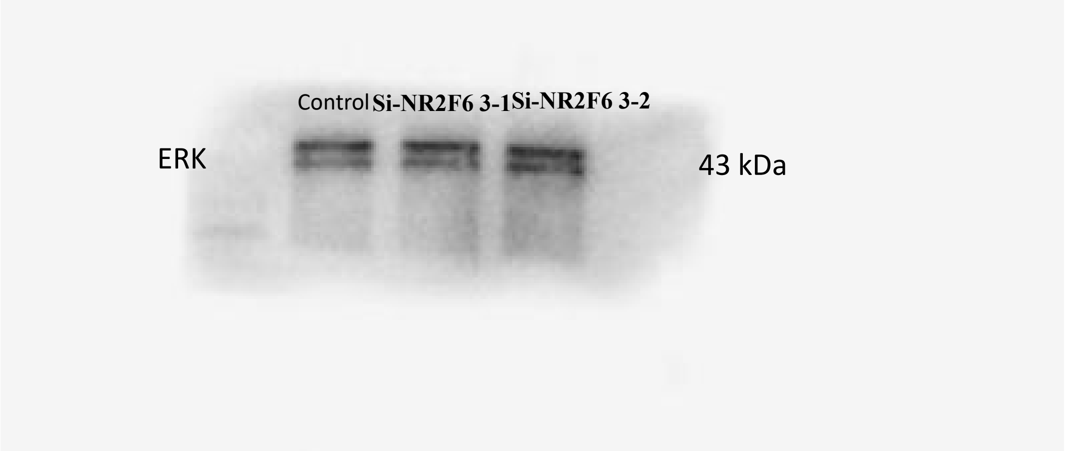
Control Si-NR2F6 3-1Si-NR2F6 3-2
ERK
43 kDa

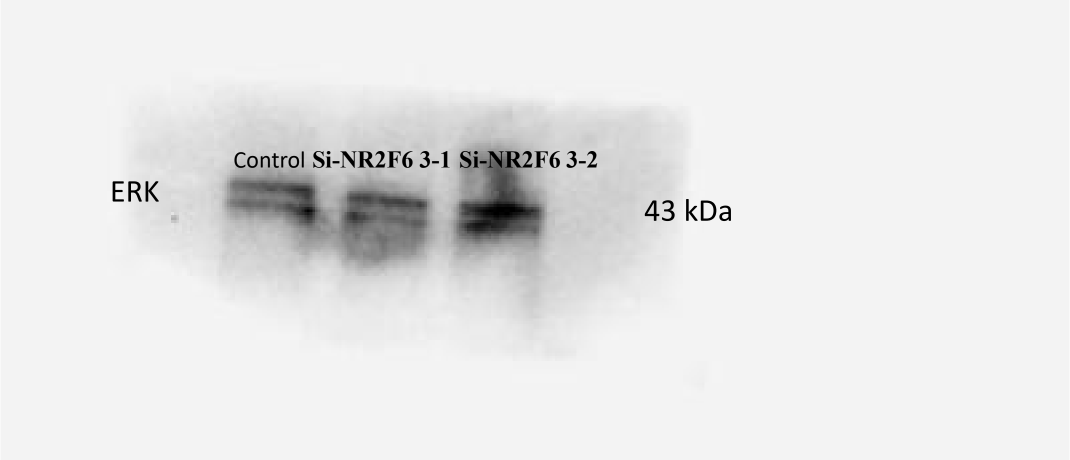
Control Si-NR2F6 3-1 Si-NR2F6 3-2
ERK
43 kDa

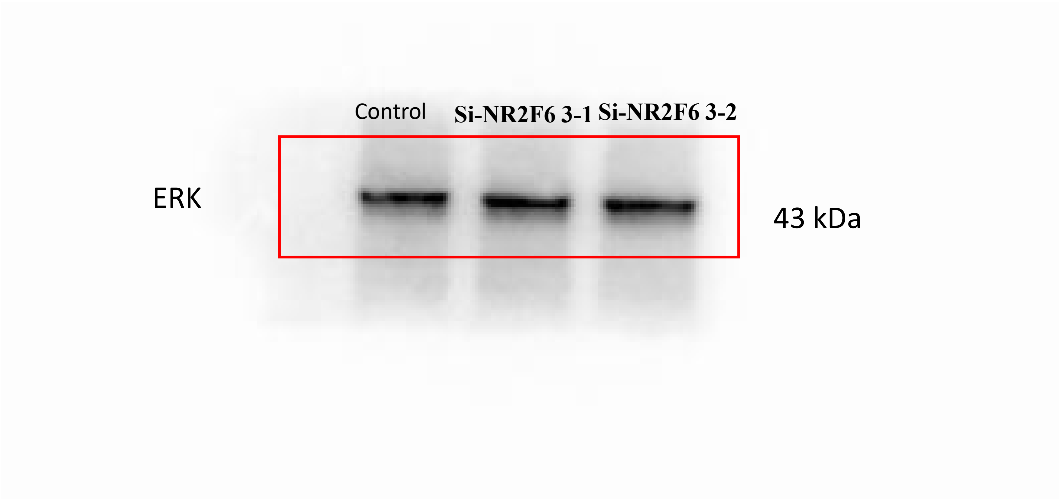
Control
Si-NR2F6 3-1
Si-NR2F6 3-2
ERK
43 kDa

GAPDH (36KDa)

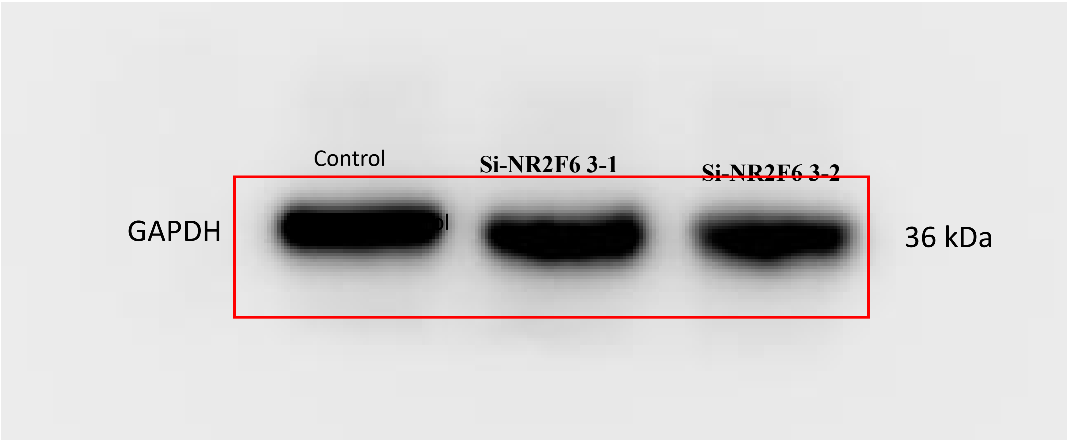
Control
Si-NR2F6 3-1
Si-NR2F6 3-2
GAPDH
36 kDa

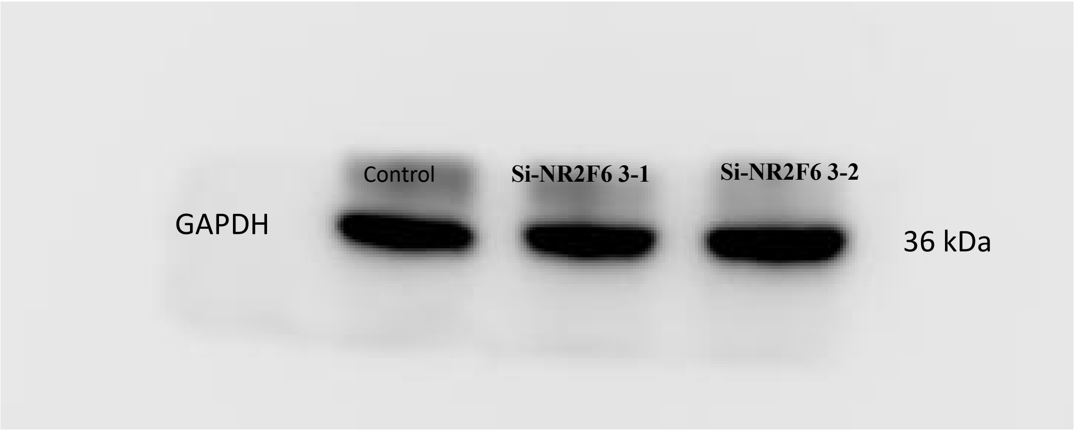
Control
Si-NR2F6 3-1
Si-NR2F6 3-2
GAPDH
36 kDa

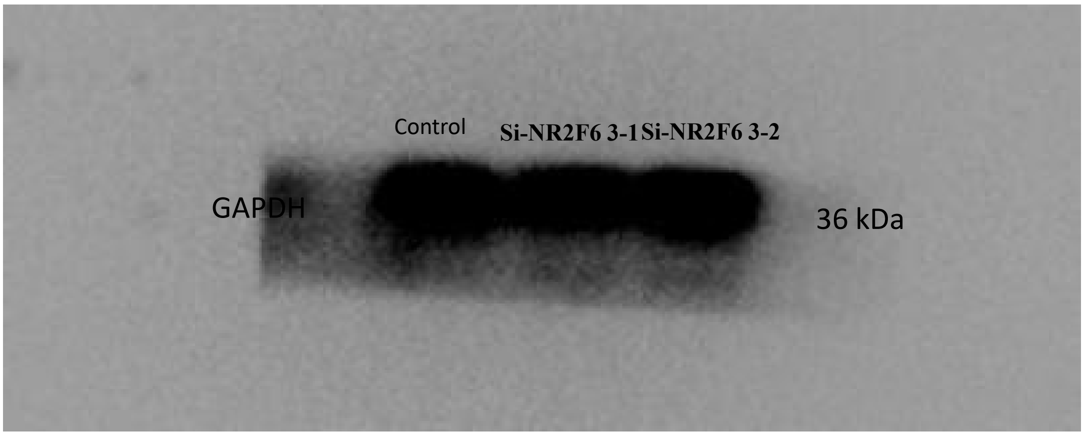
Control
Si-NR2F6 3-1
Si-NR2F6 3-2
GAPDH
36 kDa
