# Supplementary material for: NR2F6 promotes the malignant progression of neuroblastoma as an indicator of poor prognosis
Source: PLoS One. 2025 May 27;20(5):e0324334. doi: 10.1371/journal.pone.0324334 (PMC12112146; doi:10.1371/journal.pone.0324334)
Supplement: S1 Table — (PDF) [file pone.0324334.s002.pdf]

S1 Table. NR2F6 siRNA sequence

Interference	Sequence (5'-3')
h-NR2F6 3-1	sence:CGGCAAGCAUUACGGUGUCUUTT
	antisense:AAGACACCGUAAUGCUUGCCGTT
h-NR2F6 3-2	sence:CGUGGCUUUCAUGGACCAGGUTT
	antisense:ACCUGGUCCAUGAAAGCCACGTT
h-NR2F6 3-3	sence:UGAGACACUGAUCAGAGACAUTT
	antisense:AUGUCUCUGAUCAGUGUCUCATT
NC siRNA	sence:5'-UUCUCCGACAGUGUCACGU(dT)(dT) -3'
	antisense:5'-ACGUGACACUGUCGGAGAA(dT)(dT)-3'
