# Supplementary material for: NR2F6 promotes the malignant progression of neuroblastoma as an indicator of poor prognosis
Source: PLoS One. 2025 May 27;20(5):e0324334. doi: 10.1371/journal.pone.0324334 (PMC12112146; doi:10.1371/journal.pone.0324334)
Supplement: S2 Table — (PDF) [file pone.0324334.s003.pdf]

S2 Table. Sequence-specific primers

Primers	Sequence (5'-3')
NR2F6:	Forward primer: 5'-GGGGCTCTCCAGAACATC-3'
	Reverse primer: 5'-TGACACGTTGGCAGTGG -3'
GAPDH	Forward primer: 5'-GGCATTGTGGAAGGGCTCAT-3'
	Reverse primer:5'-AGATCCACGACGGACACATT-3'
