# Supplementary material for: NR2F6 promotes the malignant progression of neuroblastoma as an indicator of poor prognosis
Source: PLoS One. 2025 May 27;20(5):e0324334. doi: 10.1371/journal.pone.0324334 (PMC12112146; doi:10.1371/journal.pone.0324334)
Supplement: S3 Table — (PDF) [file pone.0324334.s004.pdf]

S3 Table. Antibodies Used for Different Experiments

Antibody	Supplier	Catalog Number	MW	WB	IHC	Immunofluorescence
GAPDH	ZENBIO	200306-7E4	36KDa	1:1000		
JNK	ZENBIO	R22866	48KDa	1:1000		
p-JNK	ZENBIO	381100	48KDa	1:1000		
ERK	ZENBIO	343830	42KDa	1:1000		
p-ERK	ZENBIO	301245	42KDa	1:1000		
p38	ZENBIO	R25239	41KDa	1:1000		
p-p38	ZENBIO	310091	41KDa	1:1000		
NR2F6	Proreintech	60117-2-lg	43KDa	1:1000	1:200	1:200
