# Supplementary material for: NR2F6 promotes the malignant progression of neuroblastoma as an indicator of poor prognosis
Source: PLoS One. 2025 May 27;20(5):e0324334. doi: 10.1371/journal.pone.0324334 (PMC12112146; doi:10.1371/journal.pone.0324334)
Supplement: S4 Table — (PDF) [file pone.0324334.s005.pdf]

S4 Table. Abbreviations and full name of pan-cancers

Abbreviations	Full name of cancers
TCGA-ACC	Adrenocortical carcinoma
TCGA-BLCA	Bladder Urothelial Carcinoma
TCGA-BRCA	Breast invasive carcinoma
TCGA-CESC	Cervical squamous cell carcinoma and endocervical adenocarcinoma
TCGA-CHOL	Cholangiocarcinoma
TCGA-COAD	Colon adenocarcinoma
TCGA-COADREAD	Colon adenocarcinoma/Rectum adenocarcinoma Esophageal carcinoma
TCGA-DLBC	Lymphoid Neoplasm Diffuse Large B-cell Lymphoma
TCGA-ESCA	Esophageal carcinoma
TCGA-FPPP	FFPE Pilot Phase II
TCGA-GBM	Glioblastoma multiforme
TCGA-GBMLGG	Glioma
TCGA-HNSC	Head and Neck squamous cell carcinoma
TCGA-KICH	Kidney Chromophobe
TCGA-KIPAN	Pan-kidney cohort (KICH+KIRC+KIRP)
TCGA-KIRC	Kidney renal clear cell carcinoma
TCGA-KIRP	Kidney renal papillary cell carcinoma
TCGA-LAML	Acute Myeloid Leukemia
TCGA-LGG	Brain Lower Grade Glioma
TCGA-LIHC	Liver hepatocellular carcinoma
TCGA-LUAD	Lung adenocarcinoma
TCGA-LUSC	Lung squamous cell carcinoma
TCGA-MESO	Mesothelioma
TCGA-OV	Ovarian serous cystadenocarcinoma
TCGA-PAAD	Pancreatic adenocarcinoma
TCGA-PCPG	Pheochromocytoma and Paraganglioma
TCGA-PRAD	Prostate adenocarcinoma
TCGA-READ	Rectum adenocarcinoma
TCGA-SARC	Sarcoma
TCGA-STAD	Stomach adenocarcinoma

TCGA-SKCM	Skin Cutaneous Melanoma
TCGA-STES	Stomach and Esophageal carcinoma
TCGA-TGCT	Testicular Germ Cell Tumors
TCGA-THCA	Thyroid carcinoma
TCGA-THYM	Thymoma
TCGA-UCEC	Uterine Corpus Endometrial Carcinoma
TCGA-UCS	Uterine Carcinosarcoma
TCGA-UVM	Uveal Melanoma
TARGET-OS	Osteosarcoma
TARGET-ALL	Acute Lymphoblastic Leukemia
TARGET-NB	Neuroblastoma
TARGET-WT	High-Risk Wilms Tumor
